# Multiplex suppression of four quadruplet codons via tRNA directed evolution

**DOI:** 10.1038/s41467-021-25948-y

**Published:** 2021-09-29

**Authors:** Erika A. DeBenedictis, Gavriela D. Carver, Christina Z. Chung, Dieter Söll, Ahmed H. Badran

**Affiliations:** 1 The Broad Institute of MIT & Harvard, Cambridge, MA USA; 2 Department of Biological Engineering, Massachusetts Institute of Technology, Cambridge, MA USA; 3 Department of Molecular Biophysics and Biochemistry, Yale University, New Haven, CT USA; 4 Department of Chemistry, Yale University, New Haven, CT USA; 5 Department of Chemistry, The Scripps Research Institute, La Jolla, CA USA

**Keywords:** Synthetic biology, Synthetic biology, tRNAs

## Abstract

Genetic code expansion technologies supplement the natural codon repertoire with assignable variants in vivo, but are often limited by heterologous translational components and low suppression efficiencies. Here, we explore engineered *Escherichia coli* tRNAs supporting quadruplet codon translation by first developing a library-cross-library selection to nominate quadruplet codon–anticodon pairs. We extend our findings using a phage-assisted continuous evolution strategy for quadruplet-decoding tRNA evolution (qtRNA-PACE) that improved quadruplet codon translation efficiencies up to 80-fold. Evolved qtRNAs appear to maintain codon-anticodon base pairing, are typically aminoacylated by their cognate tRNA synthetases, and enable processive translation of adjacent quadruplet codons. Using these components, we showcase the multiplexed decoding of up to four unique quadruplet codons by their corresponding qtRNAs in a single reporter. Cumulatively, our findings highlight how *E. coli* tRNAs can be engineered, evolved, and combined to decode quadruplet codons, portending future developments towards an exclusively quadruplet codon translation system.

## Abstract (web summary)

Genetic code expansion strategies are limited to specific codons that can be reassigned to new amino acids. Here the authors show that quadruplet-decoding tRNAs (qtRNAs) can be rapidly discovered and evolved to decode new quadruplet codons, enabling four independent decoding events in a single protein in living cells.

## Introduction

Genetic code expansion (GCE) has enabled the programmed incorporation of non-canonical amino acids (ncAAs) in proteins in living cells. This has been achieved—in nature and in the laboratory—by recoding existing redundant stop or sense codons^1–6^, increasing codon size^7–12^, or by adding additional synthetic letters to the genetic language^13,14^. These codons enable the supplementation of the proteogenic amino acid repertoire with a variety of non-proteogenic monomeric units, including noncanonical α-amino acids^15,16^, β-amino acids^17^, and benzoic and malonyl acids^18^. Although >150 ncAAs have been incorporated into proteins in vivo and in vitro^19^, cellular incorporation remains limited by the availability of readily assignable codons in a single gene^20^.

Quadruplet codons, which may enable an expanded genetic code with up to 255 unique assignable amino acids (^4^permutations = 255 quadruplet codons + 1 stop codon), may address many of the limitations of current GCE methodologies. Briefly, tRNAs containing a point insertion in the anticodon loop can often induce a + 1 frameshift during translation^21,22^. This frameshifting activity is maintained by correct Watson−Crick base pairing between bases in the tRNA anticodon and the mRNA codon^10,11,23^, enabling the faithful decoding of a four-base codon^8,12,24–27^. Recent insight into one +1 frameshifting mechanism of the *SufB2* tRNAs suggests that the first three bases of a quadruplet codon are decoded followed by a frameshift during ribosome translocation on the mRNA^22^, although other mechanisms may exist for alternative tRNAs. Whereas this process can also depend on the sequence context within the mRNA^12^, these observations suggest that it may be possible to engineer cellular protein synthesis, specifically engineering of ribosomal conformational dynamics^22^, toward sequence-specific quadruplet codon translation.

However, such broadly implemented systems have not been realized due to the unpredictable and often limited efficiency of quadruplet codon translation. Broadly, tRNA engineering may affect overall decoding efficiency through altered interactions with aminoacyl-tRNA synthetases^10,28^ and elongation factors^29–31^, perturbed ribosomal accommodation^11,32^, or limited mRNA decoding efficiency^11,32–34^. Compared to a triplet codon, translating a single quadruplet codon is substantially less efficient in unmodified, wild-type prokaryotic or eukaryotic cells; relative translational efficiencies (η, see “Methods”) of <3% are common. As translation efficiency for a protein of length N should scale with η^N^, this makes the development of an exclusively four-base codon translation system challenging. Implementing such a system will therefore require efficient, well-characterized tRNAs capable of translating quadruplet codons into the corresponding canonical amino acids (cAAs), alongside diverse ncAAs for researcher-dictated functions.

Whereas many elements of the translational apparatus have been explored for improved ncAA incorporation at quadruplet codons in biological systems, few reports have investigated quadruplet-decoding tRNAs (qtRNAs) that faithfully incorporate canonical amino acids under multiplexing conditions. Here, we develop frameworks for the discovery and characterization of amino-acid specific qtRNAs, finding that anticodon replacement in many *Escherichia coli* (*E. coli*) tRNA scaffolds can yield functional, but often inefficient, qtRNAs. We first created a system to probe quadruplet codon−anticodon interactions by nominating amino acid-specific positions in β-galactosidase (*lacZ*), yielding functional qtRNAs for many of the canonical amino acids with low suppression efficiencies (up to η = 6%). To improve qtRNA efficiency, we leveraged phage-assisted continuous evolution (qtRNA-PACE) to rapidly evolve variants with suppression efficiencies up to 40% under kinetic conditions and without genome modifications. Our most efficient qtRNAs perform on par with traditional amber suppression in cellular translation assays, can effectively compete with cellular release factors for codon decoding, show amino-acid-specific aminoacylation profiles in most cases, and allow for processive translation of up to six adjacent quadruplet codons. Furthermore, using both engineered and evolved qtRNA variants, we showcase the selective translation of a model fluorescent reporter protein using four mutually orthogonal qtRNA-quadruplet codon pairs. Collectively, our findings establish orthogonal qtRNAs for 4−20 canonical amino acids that can work in concert for the first time, and support the possibility of generating comprehensive, quadruplet-codon decoding reagents for efficient use in living cells.

## Results

### General framework for qtRNA discovery and characterization

**Fig. 1:**
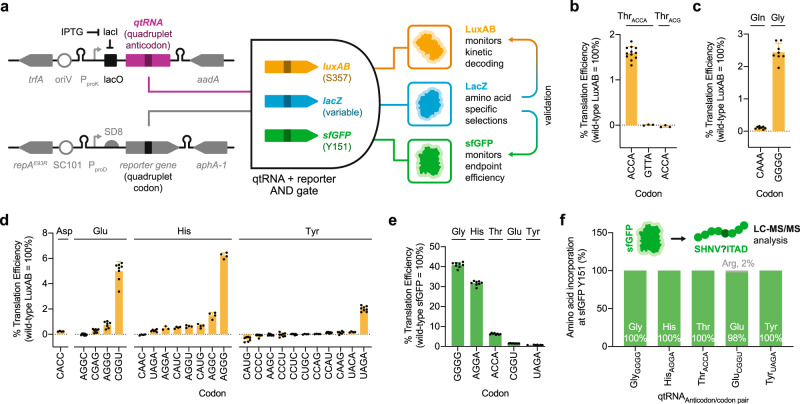
Discovery and quantification of selection derived qtRNAs. **a** Schematic representation of cellular reporter assays. Suppressed positions in the reporter proteins are shown in parentheses. In all cases, reporter protein translation prematurely terminates in the absence of a functional qtRNA due to a stop codon generated downstream of the quadruplet codon. Conversely, a functional qtRNA affects quadruplet codon suppression, yielding a full-length protein. **b** Validation of the luciferase reporter for quadruplet codon suppression. The previously described qtRNA^Thr^_ACCA_^25^ (*n* = 12 biologically independent samples) selectively decodes the cognate quadruplet codon, whereas a codon/anticodon mismatch or native triplet tRNA yields no luminescence using the quadruplet codon at residue S357 in LuxAB. Data for the remaining combinations represents the mean and standard deviation of three biologically independent samples. **c** Decoding efficiency of previously described qtRNAs, qtRNA^Gln^_CAAA_^27^, and qtRNA^Gly^_GGGG_^85^ (*n* = 8 biologically independent samples). **d** The LacZ selection pipeline yielded functional qtRNAs for aspartate (*n* = 4 biologically independent samples), glutamate (Glu *n* = 8 except for Glu-AGGG n = 7), histidine (His *n* = 4 except for His-AGGA *n* = 3 and His-UAGA *n* = 6), and tyrosine (Tyr *n* = 7 except for Tyr-CCCC, CCCU, CCAU, and UAGA *n* = 8 and Tyr-CUGC, CCAG, and UACA *n* = 4), which were assayed using the luciferase reporter. **e** Several LacZ selection-derived qtRNAs showed robust suppression of the cognate quadruplet codon at the permissive residue Y151 in sfGFP (*n* = 8 biologically independent samples, except for Tyr-UAGA *n* = 7). **f** The sfGFP reporter was used to quantify amino acid incorporation at position Y151 via mass spectrometry. Comprehensive peptide fragmentation spectra are reported in Supplementary Fig. 3 and summary LC-MS/MS results are reported in Supplementary Table 8. In all cases, reporter data is normalized to an otherwise wild-type reporter protein and color-coded by the used reporter. Unless otherwise noted, all qtRNAs were assayed against their cognate quadruplet codon incorporated at residue S357 in LuxAB or Y151 in sfGFP. Data represent the mean and standard deviation as appropriate.

To effectively monitor quadruplet decoding for our future engineering efforts, we first created a frameshift reporter by introducing a quadruplet codon into the covalently-linked bacterial luciferase LuxAB^35,36^ gene (Fig. 1a). Compared to the canonically used superfolder GFP (sfGFP), LuxAB shows a greater dynamic range which may sensitize our detection capabilities and enable the quantification of poorly active qtRNAs^35^. In addition, we monitored luminescence signals kinetically to assess qtRNA-dependent toxicity, and quantified activity using luminescence at a standard density (OD_600_) to account for differential growth rates.

Failure to decode a quadruplet codon in this reporter would lead to premature translation termination, whereas successful decoding yields full-length LuxAB and a corresponding luminescence increase. We confirmed that position 357 of LuxAB is tolerant to most amino acid substitutions (Supplementary Fig. 1a), and used it as the basis for all kinetic cellular reporter assays. To control qtRNA abundance, we designed an expression plasmid that integrates the commonly used proK promoter^37^ alongside a lacO operator to afford IPTG dependence. This yielded a comparable dynamic range to established inducible promoters (Supplementary Fig. 1b). Using this sensor, we confirmed that luminescence relies on the presence of a qtRNA and correct codon−anticodon interaction (Fig. 1b), and validated it using three previously reported qtRNAs (Fig. 1b, c and Supplementary Table 1). In all data, we unify our nomenclature by referring to qtRNAs as qtRNA^scaffold^_codon_; e.g., qtRNA^Tyr^_UAGA_ is a tyrosine tRNA scaffold containing a 5′-UCUA-3′ anticodon that should decode the cognate UAGA quadruplet codon in an mRNA.

We note that single base changes to tRNAs are known to affect their suppression activities^38^ and aminoacylation spectra^28,39–42^. In particular, tRNA anticodons often serve as identity elements for cognate aminoacyl-tRNA synthetases^39,43–46^, suggesting that anticodon engineering may alter charging fidelity. To explore quadruplet codon/anticodon interactions without inadvertently altering amino acid identity, we sought to generate a cellular reporter that may serve as the basis for selection in an amino acid-specific manner (Fig. 1a). Using *E. coli* β-galactosidase (*lacZ*), we used a degenerate library approach to first confirm that amino acids occupying several positions (D202, H392, N461, E462, Y504, and H541) are absolutely necessary for enzymatic activity (Supplementary Fig. 2a, b)^47–51^. Next, we carried out library-cross-library selections to identify functional and putatively amino-acid-specific qtRNAs. Degenerate quadruplet codon libraries at amino acid-specific positions in *lacZ* were co-transformed with a degenerate quadruplet anticodon tRNA library to nominate codon-anticodon pairs for future investigation (Supplementary Fig. 2c). In all cases, representative natural tRNA scaffolds were chosen from the *E. coli* genome for analysis in these selections (see “Methods” and Supplementary Table 2).

We identified qtRNAs that rely on a combination of previously described codon−anticodon interactions, as well as combinations that have not yet been reported (Fig. 1d and Supplementary Fig. 2c). Notably, codon−anticodon pairs derived from *lacZ* selections did not always yield measurable luminescence activity upon supplementary validation in the LuxAB reporter system. This may suggest that at least some selection-derived qtRNAs can suffer from context-dependent effects^52–55^. Nonetheless, many qtRNAs showed robust decoding efficiencies when assayed in the LuxAB reporter assay, reaching up to η = 6% (Fig. 1d).

In agreement with these data, the most functional qtRNAs show robust suppression of the cognate quadruplet codon at permissive residue Y151 in sfGFP^56^ (Fig. 1e). Mass spectrometry of sfGFP confirmed the expected amino acid profile at that position following qtRNA-dependent protein translation in the majority of cases (Fig. 1f, Supplementary Fig. 3, and Supplementary Table 8). Cumulatively, this approach independently identified 24 total qtRNAs (Supplementary Fig. 2c), of which all are novel based on to prior reports, highlighting that amino acid-specific selections can be used to nominate qtRNAs for effective quadruplet codon translation.

### Continuous directed evolution of UAGA-decoding qtRNAs

**Fig. 2:**
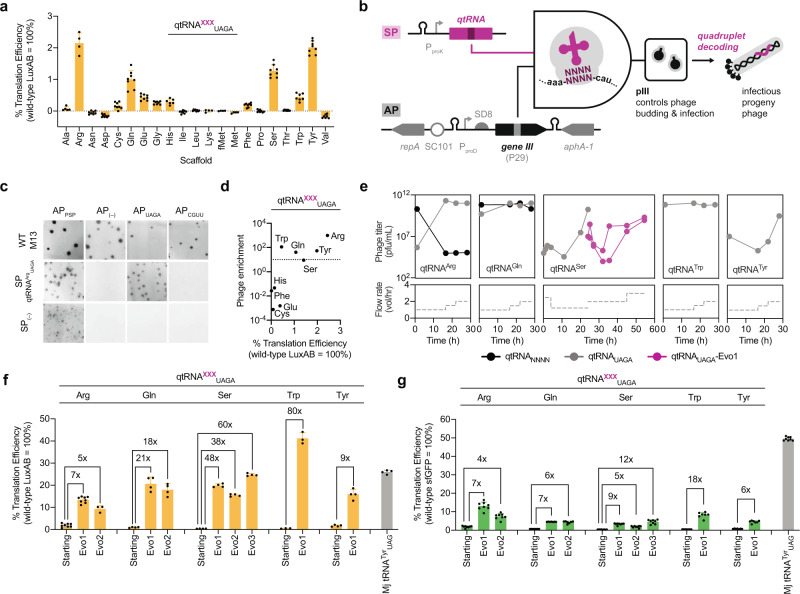
Continuous directed evolution of qtRNAs improves quadruplet codon decoding. **a** Engineered UAGA-qtRNAs using representative scaffolds for each of the 20 elongator tRNAs and initiator (fMet) tRNA validated using the LuxAB reporter assay with UAGA incorporated at residue S357; qtRNA^fMet^_UAGA_ uses a reporter with UAGA at residue 1 of LuxAB. In all data, qtRNA^XXX^_UAGA_ contains a 5′-UCUA-3′ anticodon that should decode the cognate UAGA quadruplet codon in an mRNA and “XXX” refers to the corresponding amino acid charged onto the tRNA scaffold. (*n* = 8 biologically independent samples except for Trp/Val *n* = 7; His *n* = 6; Ala *n* = 5; Arg/Lys/fMet/Met *n* = 4.) **b** Schematic representation of qtRNA-PACE circuit. Selection phages (SPs) encoding a functional qtRNA enables the translation of a quadruplet codon in *gIII* (encoded on an accessory plasmid; AP), resulting in the production of full-length pIII and infectious progeny phage. **c** SP-borne qtRNAs enable propagation as visualized by plaque assay. Mismatched qtRNA/AP pairs fail to generate phage and do not result in plaque formation. SP(–) indicates an SP lacking a qtRNA, and AP(–) indicates cells without an AP. **d** UAGA qtRNAs with detectable activities using the LuxAB reporter were tested in SP propagation assays, showing that η ≥ 0.5% are necessary to support phage enrichment above input (visualized as a dotted line). **e** Quantification of SP titers and flow rates during qtRNA-PACE campaigns using AP_1xUAGA_. Experiments were initiated with either a clonal (gray) or degenerate (black) qtRNA population, or the evolved variant Ser_UAGA_-Evo1 (purple). The second leg of qtRNA-PACE with qtRNA^Ser^_UAGA_ uses the more stringent AP_3xUAGA_. MP6 was used in all qtRNA-PACE experiments. qtRNA activities prior to (starting) and following (Evo) qtRNA-PACE campaigns and their associated fold improvements as quantified using LuxAB (**f**) or sfGFP (**g**) reporters. For LuxAB data, *n* = 4 biologically independent samples except for Arg-UAGA, and UAGA-Evo1 *n* = 8; Arg-UAGA-Evo2 *n* = 3; and Trp *n* = 3. For sfGFP data, *n* = 8 biologically independent samples except for Gln,Trp, and Tyr *n* = 7. Comprehensive sfGFP peptide fragmentation spectra for all engineered and evolved UAGA-decoding qtRNAs are reported in Supplementary Fig. 7. In all cases, reporter data is normalized to an otherwise wild-type protein and color-coded by the used reporter. Data represent the mean and standard deviation as appropriate.

Among the most functional qtRNAs identified through the *lacZ* library-cross-library selection, we noted that the qtRNA^Tyr^_UAGA_ and qtRNA^His^_UAGA_ showed particularly low activity (Fig. 1d). Building on previous studies that have repurposed UAGA-decoding qtRNAs, we sought to further explore and improve the efficiency of UAGA-decoding in an unbiased manner. To begin, we chose a unique *E. coli* elongator tRNA for each of the 20 canonical amino acids alongside an initiator tRNA^Met^ (see “Methods”, Supplementary Table 2) to serve as scaffolds for qtRNA engineering. In each scaffold, we substituted the anticodon with the sequence 5′-UCUA-3′ to enable UAGA quadruplet codon decoding. We characterized the quadruplet translation efficiency of the 21 engineered UAGA-decoding qtRNAs, finding that nearly half of these qtRNAs (10/21) demonstrated modest UAGA decoding activity compared to triplet decoding (η ≤ 2.5%; Fig. 2a). Universally, the engineered UAGA qtRNAs did not cause appreciable host fitness defects (Supplementary Table 3).

As UAGA-decoding qtRNAs repurpose the low-usage UAG stop codon^57^, they may experience competition with release factor 1 (RF1) for efficient suppression^33,58,59^. Although RF1 deletion can improve UAGA decoding, the resultant strains can show significant fitness defects in rich media^59^, spontaneous reversions to correct genomic instabilities^60^, and low amino acid incorporation fidelity at targeted UAG codons^61^. To circumvent these limitations, we hypothesized that supplementary qtRNA modifications may improve UAGA quadruplet codon translation efficiency. Indeed, tRNA scaffold mutations, with a particular focus on stem engineering, can play a key role in the development of host-tolerated and efficient orthogonal tRNAs^62^. These may occur via optimization of scaffold-anticodon compatibility^63,64^, by improving qtRNA interactions that were affected by anticodon engineering^65^, or through enhanced competition with RF1.

To improve qtRNA activities in an unbiased manner, we developed a directed evolution platform based on phage-assisted continuous evolution (PACE)^66^ (Fig. 2b). Briefly, a qtRNA is encoded on a selection phage (SP) in place of the M13 bacteriophage minor coat gene *gIII* (translated to pIII). Following infection, the qtRNA may suppress a quadruplet codon in *gIII* encoded by an accessory plasmid (AP), resulting in complementation of the *gIII*-deficient SP and robust progeny propagation. To enhance qtRNA genetic diversity, cellular mutation rates are enhanced by overexpression of mutagenesis plasmid (MP6)-borne mutator proteins^67^.

We introduced a single quadruplet codon at the permissive residue P29 of pIII^35^ in the AP, thereby generating an amino acid-independent selection for all qtRNAs. We validated this system by challenging *E. coli* cells bearing APs encoding a CGUU or UAGA quadruplet codon in pIII with SPs encoding or lacking the corresponding qtRNA^Arg^_UAGA_, our most active UAGA variant. SP-qtRNA^Arg^_UAGA_ showed robust translation of pIII (visualized as viral plaques) using AP_UAGA_ but not AP_CGUU_, and SPs lacking a qtRNA did not show any visible plaques (Fig. 2c). We extended this analysis to additional UAGA qtRNAs, finding a strong correlation between luciferase output and SP propagation in liquid culture (Fig. 2d).

Using this system, we evolved the top five engineered qtRNAs (qtRNA^Gln^_UAGA_, qtRNA^Arg^_UAGA_, qtRNA^Ser^_UAGA_, qtRNA^Trp^_UAGA_, and qtRNA^Tyr^_UAGA_) and two degenerate qtRNA libraries (qtRNA^Gln^_NNNN_ and qtRNA^Arg^_NNNN_) for 28 h of qtRNA-PACE using AP_1xUAGA_ and MP6^67^ (Fig. 2e, and Supplementary Fig. 6). By the end of this short campaign, all qtRNAs retained or discovered the expected 5′ UCUA 3′ anticodon, and evolved qtRNAs showed improved SP propagation activities compared to their starting counterparts (Supplementary Figure 4).

We subcloned each unique evolved qtRNAs into expression plasmids and assayed quadruplet codon translation efficiencies using the LuxAB reporter, finding that all qtRNAs had improved by nearly an order of magnitude in quadruplet codon decoding efficiency (10% < η < 40%) without corresponding increases in toxicity (Fig. 2f; Supplementary Table 3). We note qtRNA^Ser^_UAGA_ evolution yielded a previously characterized double mutant (qtRNA^Ser^_UAGA_-Evo1; C32A, A38C) with improved quadruplet codon suppression activity^8^. Evolution of qtRNA^Ser^_UAGA_-Evo1 SP for an additional 30 h of qtRNA-PACE (Fig. 2e) using the more stringent AP_3xUAGA_ and MP6 gave rise to qtRNA^Ser^_UAGA_-Evo2 and qtRNA^Ser^_UAGA_-Evo3 (Supplementary Fig. 6c). Both variants supported SP propagation using APs that encode up to four UAGA codons (Supplementary Fig. 4) and moderately improved activities in the LuxAB reporter (Fig. 2f).

Whereas qtRNA-dependent translation has historically been less efficient than amber (UAG) suppression strategies, our evolved variants showed efficiencies comparable to the commonly used wild-type *Methanocaldococcus jannaschii* TyrRS-tRNA^Tyr^_UAG_ pair^68^ when validated in the luciferase reporter (Fig. 2f), and a slight reduction in efficiency when compared using the sfGFP reporter (Fig. 2g). It should be noted that absolute comparisons between the sfGFP and LuxAB reporters may offer limited insight due to differences in quadruplet codon sequence context, local mRNA secondary structure, reporter gene length, and/or the nature of reporter activity. Further assaying engineered and evolved UAGA qtRNAs against an sfGFP reporter incorporating a UAGA codon at residue Y151^56^ in two different RF1 knockout strains (C321.ΔA^59^ and JX33^69^) showed robust decoding activity (η ≤ 75%; Supplementary Fig. 5). Collectively, these findings agree with prior work that RF1 competition can limit the efficiency of UAGA-qtRNAs^33^, and highlight the ability of qtRNA-PACE to rapidly evolve qtRNAs with greatly improved quadruplet codon decoding capabilities in vivo.

### Analysis of qtRNA aminoacylation fidelity

Mutations acquired through qtRNA-PACE campaigns occurred in several regions of the qtRNA (Supplementary Fig. 6), including interaction interfaces with cognate aminoacyl-tRNA synthetases (aaRSs)^70^. We hypothesized that these mutations may improve quadruplet codon translation by affecting aminoacylation efficiency by the cognate aaRS, or through recruitment of a non-cognate aaRS following the adoption of key identity elements. To investigate this possibility, we leveraged the aforementioned sfGFP reporter by incorporating a UAGA codon at residue Y151^56^ and quantified amino acid occupancy via mass spectrometry (Fig. 1f). We find that the expected amino acid was incorporated in both pre- and post-evolution qtRNA^Gln^_UAGA_, qtRNA^Arg^_UAGA_, qtRNA^Ser^_UAGA_, and qtRNA^Tyr^_UAGA_ variants, with <0.4% contaminating amino acid (Supplementary Fig. 7, Supplementary Table 4, and Supplementary Table 8).

**Fig. 3:**
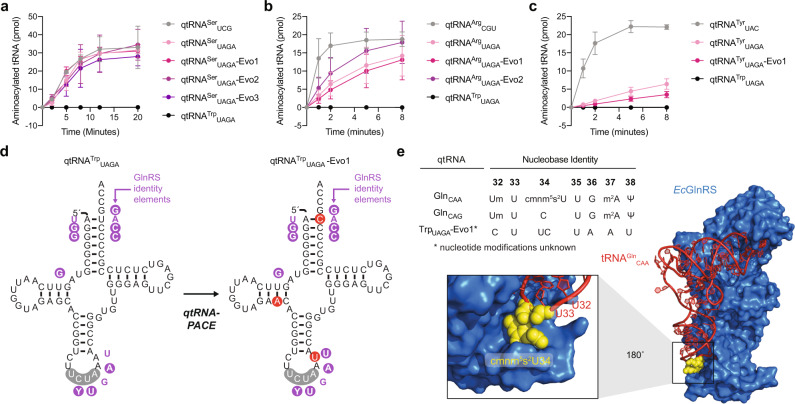
In vitro analysis of evolved qtRNAs. In vitro aminoacylation of qtRNAs using *Ec*SerRS (**a**), *Ec*ArgRS (**b**), and *Ec*TyrRS (**c**); qtRNA^Trp^_UAGA_ was used as a negative control in all cases. **d** Analysis of *Ec*GlnRS identity elements as found in the engineered qtRNA^Trp^_UAGA_ and qtRNA-PACE evolved qtRNA^Trp^_UAGA_-Evo1. **e** Anticodon and adjacent nucleobase identities for known *E. coli* glutamine tRNAs and the qtRNA-PACE evolved qtRNA^Trp^_UAGA_-Evo1. Position of the modified nucleotide cmnm^5^s^2^U (yellow) in the co-crystal structure of tRNA^Gln^_CAA_ and *E. coli* GlnRS (4JYZ; https://www.wwpdb.org/pdb?id=pdb_00004jyz). Data represent the mean and standard deviation of three biological replicates.

Since the mutations did not affect misacylation by non-cognate aaRSs in vivo, we hypothesized that they may improve the catalytic efficiency of aminoacylation by the cognate aaRS in some cases. We accordingly measured the aminoacylation kinetics of qtRNA^Arg^_UAGA_, qtRNA^Ser^_UAGA_, and qtRNA^Tyr^_UAGA_ variants by their cognate *E. coli* aminoacyl-tRNA synthetases in vitro^36^ (Fig. 3a−c). Whereas all serine qtRNAs are aminoacylated by *Ec*SerRS with comparable efficiencies, qtRNA^Arg^_UAGA_ and qtRNA^Tyr^_UAGA_ variants show moderately abrogated aminacylation kinetics by their cognate aaRSs (*Ec*ArgRS and *Ec*TyrRS) as compared to cognate wild-type triplet-decoding tRNAs. As all evolved variants show comparable activities in vivo, these findings may suggest that aminoacylation kinetics are not limiting for quadruplet codon translation in *E. coli* under the tested conditions.

In contrast, anticodon engineering of qtRNA^Trp^_UAGA_ resulted in a qtRNA predominantly misacylated with glutamine (81.7% Gln, 5.9% Trp, 12.4% Tyr) (Supplementary Table 4). Our qtRNA-PACE campaign gave rise to three additional base changes: the loss of base pairing at both U12•G24 (G24A) in the D-loop and A1•U72 (U72C) in the acceptor stem, and A38U in the anticodon loop (Fig. 3d). These changes, in particular A38U which is a known *Ec*GlnRS identity element^71^, ensure that qtRNA^Trp^_UAGA_-Evo1 is nearly exclusively misacylated with glutamine (99.7% Gln, 0.03% Tyr).

Furthermore, tRNA^Trp^ and tRNA^Gln^ scaffolds are known to switch identity by a C35U substitution in the anticodon^72^. Through examination of the *Ec*GlnRS-tRNA co-crystal structure (PDB: 1GSG^73^), we noted that tRNA^Gln^_CAA_ contains the hypermodified nucleotide 5-carboxyaminomethyl-2-thiouridine (cmnm^5^s^2^U) at anticodon position 34^74^ (Fig. 3e). Given that qtRNA^Trp^_UAGA_-Evo1 is an *Ec*GlnRS substrate, it is possible that the first two bases of the quadruplet anticodon 5′ UCUA 3′ are accommodated in the space allotted to cmnm^5^s^2^U34, allowing the third base of the quadruplet anticodon (U) to occupy position 35. These results suggest that qtRNAs will often evolve along trajectories that maintain the aminoacylation profiles of the engineered variants. Future engineering efforts must therefore ensure specific aminoacylation by the cognate synthetase prior to evolution using qtRNA-PACE.

### Characterization of context-dependence and processivity of evolved qtRNAs

**Fig. 4:**
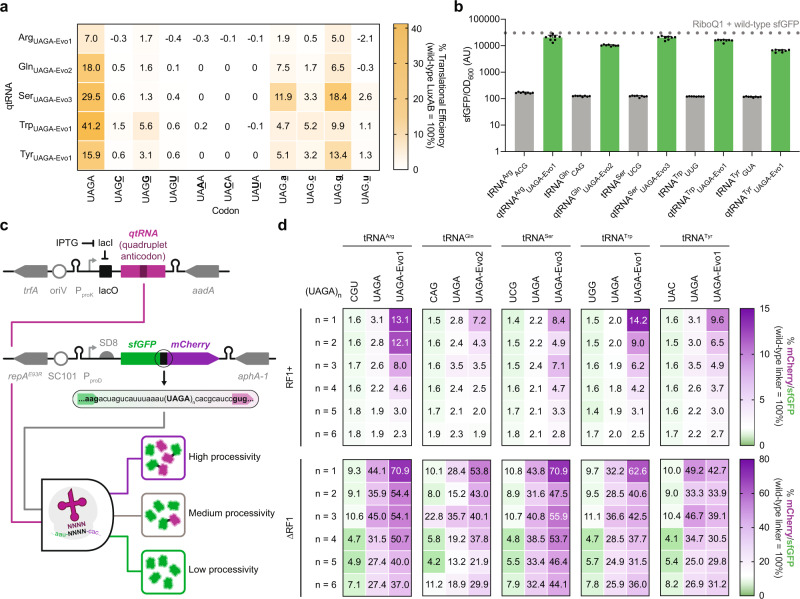
Quantification of evolved qtRNA crosstalk and processivity. **a** Evolved UAGA-qtRNAs show moderate crosstalk with codons bearing a different nucleotide at the fourth position but not the third position of the quadruplet codon. Evolved UAGA-qtRNAs can additionally crosstalk with amber (UAG) codons, leading to a frameshift that is dependent on the base following the stop codon. In all cases, the activity of non-cognate codon-anticodon pairs is lower than the expected cognate interactions. **b** Quadruplet-codon translation using a previously described orthogonal ribosome RiboQ1 (contains mutations U531G, U534A, A1196G, A1197G in the 16S rRNA)^11^ (*n* = 8 biologically independent samples). **c** Schematic representation of the dual sfGFP-mCherry reporter assay to investigate the processivity of quadruplet codon translation. In all cases, sfGFP and mCherry are separated by a linker composed of adjacent UAGA quadruplet codons. The ratio of mCherry to sfGFP is a proxy for UAGA codon suppression efficiency and processivity. **d** Evolved UAGA qtRNAs effectively translate linkers containing up to 5−6 adjacent UAGA quadruplet codons in the RF1 + strain S3489 (top) or the ∆RF1 strain JX33 (bottom). Although showing moderate efficiencies, these tandem suppression events have been validated using mass spectrometry (wherever possible) and comprehensive peptide fragmentation spectra are reported in Supplementary Fig. 10, and summary LC-MS/MS results are reported in Supplementary Table 8. In all cases, reporter data is normalized to an otherwise wild-type protein and color-coded by the used reporter. Data represent the mean and standard deviation as appropriate.

We next explored codon-anticodon crosstalk of PACE-evolved qtRNAs, as canonical tRNAs often decode both cognate and wobble codons. To investigate whether a comparable relationship exists for evolved qtRNAs, we characterized their decoding specificity using our sensitized LuxAB reporter with variations at the third or fourth position of the quadruplet codon (Fig. 4a). Whereas third position variations resulted in ablated luminescence, codons containing a mismatched fourth base were moderately translated, in agreement with prior findings^23,26,75^ (Supplementary Fig. 8). During stringent qtRNA-PACE selections, we noticed the SP genome of qtRNA^Ser^_UAGA_ evolved UAG codons within the highly expressed phage gene *gVIII*, suggesting that qtRNAs may crosstalk triplet codons in vivo. Indeed, we find that crosstalk with UAG triplet codons is more prevalent than crosstalk with other near-cognate quadruplet codons (Fig. 4a and Supplementary Fig. 8). This UAG triplet codon crosstalk and resultant frameshift may therefore preclude the use of UAG and UAGN suppressors concurrently. Interestingly, all tested qtRNAs exhibited similar crosstalk trends, suggesting a unifying mechanism to decoding UAGA codons.

Furthermore, all evolved qtRNAs can decode UAGA codons during translation using an orthogonal ribosome (Fig. 4b). Interestingly, incorporation of rRNA mutations that improve quadruplet codon translation (RiboQ1: U531G/U534A/A1196G/A1197G)^11^ showed similar efficiency as wild-type host ribosomes (Supplementary Fig. 9), suggesting that further ribosomal evolution may be necessary to globally enhance quadruplet codon decoding. We hypothesize that future evolution campaigns may integrate an orthogonal ribosome to improve cellular decoding efficiencies

Another factor limiting the broad implementation of quadruplet codon translation has been poor processive translation of multiple quadruplet codons, largely due to low qtRNA translation efficiency at single quadruplet codons. To minimize any context-dependence and further investigate the processivity of quadruplet codon translation, we constructed a dual reporter protein-encoding sfGFP and mCherry separated by a linker composed of adjacent UAGA quadruplet codons (Fig. 4c). The efficiency of quadruplet codon translation and processivity can be easily quantified by comparing the relative signal intensities of both fluorescent proteins (Fig. 4d). This strategy has previously been employed to quantify UAG readthrough in eukaryotic cells^76^ and a similar dual fluorescence reporter system has been used to study translational errors and stop codon readthrough in *E. coli*^77^.

We found that optimized variants qtRNA^Ser^_UAGA_-Evo3, qtRNA^Arg^_UAGA_-Evo1, qtRNA^Tyr^_UAGA_-Evo1, qtRNA^Trp^_UAGA_-Evo1, and qtRNA^Gln^_UAGA_-Evo2 were able to translate a linker containing up to six adjacent UAGA quadruplet codons (Fig. 4d and Supplementary Fig. 10). Analysis of the same qtRNA set using the linked sfGFP-mCherry reporters assayed in the JX33 (∆RF1) strain revealed a greater increase in quadruplet codon decoding processivity, but showed a higher level of readthrough in the absence of the requisite qtRNAs (Fig. 4d). Similar background translation has been noted in ∆RF1 strains in part due to the incorporation of triplet codon-encoded canonical amino acids^33^.

### Suppression of four unique, orthogonal quadruplet codons in sfGFP

**Fig. 5:**
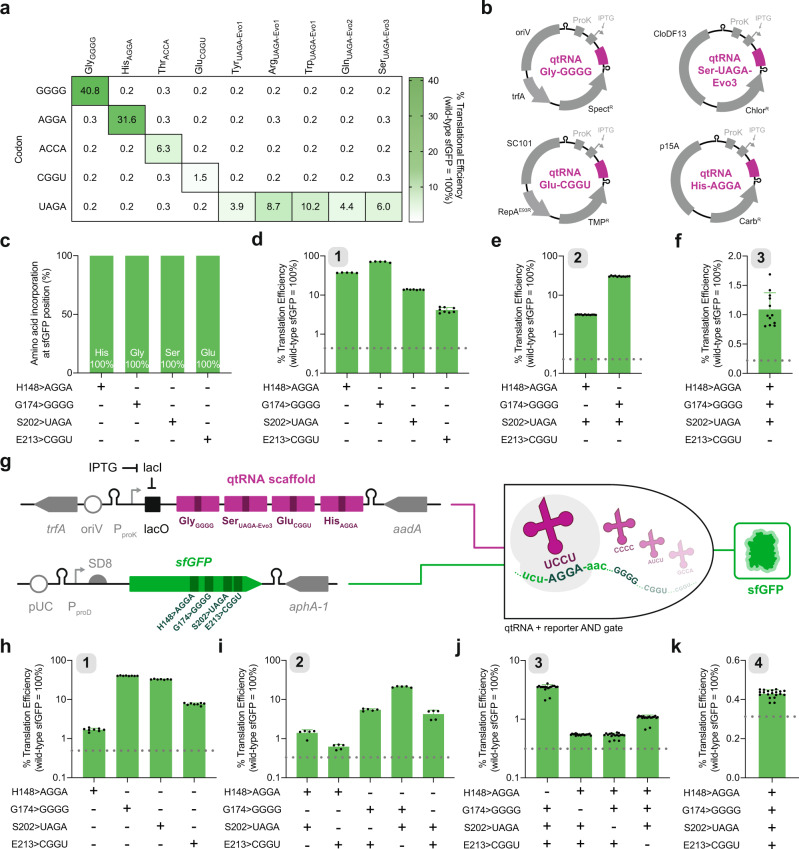
Multiplex quadruplet codon suppression in sfGFP. **a** Engineered and evolved qtRNAs showcase robust mutual orthogonality position Y151 of sfGFP. Expected codon-qtRNA interactions are outlined. **b** Schematic representation of orthogonal qtRNA expression plasmid architecture, including qtRNA, plasmid origin, and antibiotic resistance markers. **c** The sfGFP reporter previously described was first used to quantify amino acid incorporation at positions throughout sfGFP via mass spectrometry. Comprehensive peptide fragmentation spectra are reported in Supplementary Figure 11 and summary LC-MS/MS results are reported in Supplementary Table 8. Using a reporter with unique quadruplet codons in sfGFP alongside orthogonal qtRNA expression plasmids encoding qtRNA^Gly^_GGGG_, qtRNA^Ser^_UAGA_-Evo3, qtRNA^Glu^_CGGU_, and/or qtRNA^His^_AGGA_ enables the translation of one (*n* = 5 biologically independent samples except for S202 > UAGA *n* = 7 and E213 > CGGU *n* = 8) (**d**), two (*n* = 12 and n = 11 biologically independent samples, respectively) (**e**), and three (*n* = 12) (**f**) unique quadruplet codons in a single reporter gene. **g** Schematic representation of the engineered multicistronic qtRNA scaffold and reporter plasmid encoding multiple quadruplet codons in sfGFP. Production of full-length sfGFP is dependent on the translation of up to four quadruplet codons. Expression of qtRNA^Gly^_GGGG_, qtRNA^Ser^_UAGA_-Evo3, qtRNA^Glu^_CGGU_, and qtRNA^His^_AGGA_ from a single multicistronic qtRNA scaffold enables the translation of one (*n* = 8 biologically independent samples except for S202 > UAGA n = 7) (**h**), two (*n* = 5 biologically independent samples) (**i**), three (*n* = 18 biologically independent samples except for H148 > AGGA/G174 > GGGG/S202 > UAGA *n* = 20) (**j**) and four (*n* = 20 biologically independent samples) (**k**) quadruplet codons in sfGFP. Comprehensive peptide fragmentation spectra are reported in Supplementary Figs. 14–17. In all cases, reporter data is normalized to an otherwise wild-type protein and color-coded by the used reporter. Dotted lines on each plot represent strain autofluorescence and gray boxes in the upper left corner of certain plots reflect number of quadruplet codons. Data represent the mean and standard deviation as appropriate.

To enable extensive quadruplet codon translation, qtRNAs with efficient and mutually orthogonal decoding capabilities are needed. We, therefore, leveraged a previously described qtRNA (qtRNA^Gly^_GGGG_), variants discovered through *lacZ* selections (qtRNA^His^_AGGA_, qtRNA^Glu^_CGGU_), and the most active UAGA-qtRNAs evolved in PACE (qtRNA^Tyr^_UAGA_-Evo1, qtRNA^Arg^_UAGA_-Evo1, qtRNA^Trp^_UAGA_-Evo1, qtRNA^Gln^_UAGA_-Evo2, and qtRNA^Ser^_UAGA_-Evo3) to investigate their mutual orthogonality. Using sfGFP with the corresponding quadruplet codons at position Y151, we find exceptional degrees of orthogonality between all tested qtRNAs (Fig. 5a).

Emboldened by these findings, we explored the ability of four qtRNAs to function alongside one another and translate sfGFP through multiplexed decoding of cognate quadruplet codons. To permit the exploration of >2 decoding events in a single reporter, we developed a series of qtRNA expression plasmids encoding mutually orthogonal origins of replication and resistance markers, enabling their concurrent use in a single *E. coli* cell (Fig. 5b). For all assays, we used the most active UAGA-qtRNAs based on faithful amino acid incorporation (Supplementary Table 4). We first confirmed exclusive incorporation of the expected amino acids at various positions throughout sfGFP (Fig. 5c and Supplementary Fig. 11). Using these orthogonal plasmids, we were able to translate one (η = 4−37%; Fig. 5d), two (η = 3−30%; Fig. 5e), and three (η = 1%; Fig. 5f) unique quadruplet codons in sfGFP. On average, trends in the observed qtRNA decoding efficiencies aligned moderately with plasmid copy number (Supplementary Fig. 12), suggesting that they may be constrained by qtRNA abundance upon induction as previously noted^78^.

Our attempts to extend these results to four unique qtRNA expression plasmids showed fitness defects in the resultant strains, likely due to plasmid load on the cell (Supplementary Table 5). To minimize the number of orthogonal plasmids necessary to decode more than three events in a single reporter, we hypothesized that we could create single plasmids capable of expressing multiple unique qtRNAs via multicistronic cassettes. To obviate the creation of highly repetitive synthetic qtRNA operons, we instead mined endogenous multicistronic tRNA operons from *E. coli* and chose six unique scaffolds that contain at least four tRNAs each as the basis for our synthetic constructs (Supplementary Table 6). Guided by qtRNA decoding efficiency in LuxAB and sfGFP assays, we repurposed these six scaffolds to include qtRNAs in descending order of activity: qtRNA^Gly^_GGGG_, qtRNA^Ser^_UAGA_-Evo3, qtRNA^Glu^_CGGU_, then qtRNA^His^_AGGA_ (Fig. 5g and Supplementary Table 7). Of the six qtRNA scaffold assayed, scaffold #2 showed the highest overall percent translational efficiency (Supplementary Fig. 13). Further interrogation of qtRNA scaffold #2 showed efficient decoding of one (η = 1.5−40%; Fig. 5h), two (η = 0.6−22%; Fig. 5i), and three (η = 0.5−4%; Fig. 5j) quadruplet codons in sfGFP. Excitingly, we find that qtRNA scaffold #2 also enables translation of four unique quadruplet codons (η = 0.45%) in sfGFP (H148 > AGGA, G174 > GGGG, S202 > UAGA, and E213 > CGGU) in a qtRNA-dependent manner (Fig. 5k). Specific amino acid incorporation corresponding to the translation of three (Supplementary Figs. 14, 15) and four quadruplet codons (Supplementary Figs. 16, 17) in sfGFP were validated via protein purification and mass spectrometry (Supplementary Table 8). Taken together, these results demonstrate the first successful attempt at decoding four unique quadruplet codons in living cells.

## Discussion

Genetic code expansion methods have historically been limited by low efficiency^11,32–34^, competition with host cellular factors^28–30^, and the need for whole-genome codon reassignment^1–5^. To circumvent these issues, we first developed a selection scheme using *E. coli* β-galactosidase to nominate functional and amino acid-specific codon-anticodon pairs that could serve as robust starting points for further optimization (Fig. 1a). To improve quadruplet codon−decoding capabilities of qtRNAs corresponding to canonical amino acids, we developed a system for the unbiased, phage-assisted continuous evolution of qtRNA scaffolds beyond the anticodon and flaking sequences (Fig. 2b). By evolving five unique UAGA-decoding qtRNAs, we noted a general trend wherein mutations immediately flanking the anticodon were acquired and greatly improved their activities (Fig. 2f−g and Supplementary Table 4). Using these nominated qtRNAs, we sought to further investigate context-dependence and processivity (Figs. 3–4). Finally, towards the extensible development of an in vivo quadruplet codon-based translation system, we utilized multicistronic tRNA operons allowing four qtRNAs aminoacylated by endogenous synthetases to function in concert to simultaneously decode up to four mutually orthogonal quadruplet codon decoding events in a single reporter for the first time (Fig. 5 and Supplementary Table 6).

The development of a plasmid-based system that capitalizes on endogenous *E. coli* translational machinery, confers minimal host fitness defects, and does not require extensive strain engineering overcomes major limitations of current genetic code expansion technologies. Using our discovered and evolved qtRNAs, we explored key properties essential for a quadruplet codon-based genetic code, including characterization of quadruplet codon specificity and crosstalk, and UAGA-decoding qtRNA competition with RF1 (Figs. 3, 4d). We showed that qtRNAs crosstalk with quadruplet codons that mismatch at the fourth position, quantified a previously unidentified form of crosstalk with related triplet codons (Fig. 4a), and illustrated the efficient use of a previously described orthogonal ribosome to enable quadruplet-codon translation (Fig. 4b). Investigating qtRNA competition with RF1 wherein engineered UAGA-qtRNAs repurpose the low-usage UAG stop codon, we illustrate increased qtRNA suppression activity in a RF1 knockout strain (Fig. 4d). This not only showed effective competition with RF1, but also that evolved qtRNAs have strain-independent suppression improvements. Furthermore, we demonstrated processive protein translation using a non-canonical genetic code, a corollary of the high efficiency of our qtRNAs, and an essential feature for future quadruplet codon decoding (Fig. 5). Notably, qtRNA-PACE-derived mutations in the anticodon flanking sequences that increased suppression activity may suggest the existence of general patterns that govern quadruplet anticodon efficiency^79^, as have been previously described for triplet codons^63^, and providing a systematic route to improve qtRNA activities.

Cumulatively, our findings highlight significant qtRNA evolvability for efficient and amino acid-specific quadruplet codon translation, demonstrating essential properties necessary for the development of an exclusively quadruplet codon code. Through the extension of this directed evolutionary framework and the newly developed orthogonal multi-qtRNA expression plasmids, rRNA engineering^80^ and evolution strategies may be necessary to eliminate crosstalk with triplet codons and maintain quadruplet coding frames. In particular, targeted engineering of ribosomal conformational may further improve decoding fidelity beyond the recently described +1 frameshifting during tRNA-mRNA translocation^22^. Additionally, we expect that our designed multicistronic qtRNA cassettes, alongside methods for ncAA incorporation and negative selection during continuous evolution^35^, will allow for the simultaneous directed evolution of dedicated qtRNA-synthetase pairs for canonical and/or non-canonical amino acids. This work therefore bolsters the toolkit available to synthetic biologists investigating genetic code expansion in vivo, and may serve as the basis for the creation of de novo quadruplet codon/qtRNA pairs for each of the 20 canonical amino acids.

## Methods

### General methods

Antibiotics (Gold Biotechnology) were used at the following working concentrations unless otherwise noted: carbenicillin, 50 μg/mL; spectinomycin, 100 μg/mL; chloramphenicol, 40 μg/mL; kanamycin, 30 μg/mL; tetracycline, 10 μg/mL; streptomycin, 50 μg/mL. David Rich Medium (DRM)^20^ was used for PACE and all experiments involving plate reader measurements, excluding experiments involving JX33 RF1 knockout strain. For all other purposes, including phage-based selection assays, general cloning, and all experiments involving JX33 RF1 knockout strain, 2xYT media was used. All PCRs were performed using Phusion U HotStart DNA Polymerase (Life Technologies). Key plasmids from this study have been deposited on Addgene. See the Extended Supplement for all plasmids and plasmid maps, Addgene links, catalog numbers of materials, and plasmids used to produce each figure.

### Chemically competent cell preparation

Strain S3489, a K12 derivative of S2060^81^ further optimized for directed evolution by deletion of ribosome hibernation-promoting factor Hpf, was used in all reporter assays, phage propagation assays, plaque assays, and PACE campaigns unless otherwise noted. To prepare competent cells, an overnight culture was diluted 1,000-fold into 50 mL of 2xYT media supplemented with maintenance antibiotics and grown at 37 °C with shaking at 230 × *g* to OD_600_ ~0.4–0.6. Cells were pelleted by centrifugation at 6,000× RCF for 10 min at 4 °C. The cell pellet was then resuspended by gentle stirring in 5 mL of TSS (LB media supplemented with 5% v/v DMSO, 10% w/v PEG 3350, and 20 mM MgCl_2_). The cell suspension was stirred to mix completely, aliquoted, and flash-frozen in liquid nitrogen, and stored at −80 °C until use.

### USER cloning

Plasmids were cloned using USER (Uracil-Specific Excision Reagent) assembly, wherein primers were designed to include a USER junction, denoting the region between the 5′ primer end containing a dA and a deoxyuracil base approximately 15 base pairs downstream. USER junctions were additionally designed to have a 55 °C < *T*_*m*_ < 60 °C and minimal secondary structures. PCR products were run on a 1% agarose gel containing approximately 0.2 µg/mL ethidium bromide, allowing visualization under ultraviolet light, and subsequently purified using QIAquick Gel Extraction kit (Qiagen). Fragments were quantified using a NanoDrop 1000 Spectrophotometer (Thermo Fisher Scientific). Fragments containing complementary USER junctions were added in an equimolar ratio of between 0.2 and 1 pmol to a 10 µl reaction containing 1 µl CutSmart Buffer (50 mM potassium acetate, 20 mM Tris-acetate, 10 mM magnesium acetate, 100 µg/mL BSA at pH 7.9; New England Biolabs), 0.75 µL DpnI (New England Biolabs), and 0.75 µL USER enzyme (Uracil-DNA Glycosylase and DNA-glycosylase-lyase Endonuclease VIII; New England Biolabs). Reactions were incubated at 37 °C for 20 min, then heated to 80 °C for 3 min, and slowly cooled to 12 °C at 0.1 °C/s. During this assembly, uracil DNA-glycosylase catalyzes the excision of a dU, creating an apyrimidinic site at which Endonuclease VIII breaks the phosphodiester backbone. Assembled constructs were added to 100 µL 2x KCM (100 mM KCl, 30 mM CaCl_2_, 50 mM MgCl_2_ in MilliQ H_2_O) and 100 µL competent cells. For all cloning, we used either Mach1F (Mach1 T1^R^ cells (Thermo Fisher Scientific) mated with F’ episome of S2060 strain^31^), NEB Turbo (New England Biolabs), DH5α (Thermo Fisher Scientific), or 10-beta (New England Biolabs) cells. Cells were flicked to mix and incubated on ice for 10 min, heat shocked at 42 °C for 1.5 min, and then placed back on ice for 2 min. Cells were allowed to recover in 1 mL 2xYT at 37 °C with shaking between 230 and 300 × *g* for at least 45 min. Cells were then streaked on 1.5% agar-2XYT supplemented with appropriate antibiotics and incubated at 37 °C for 16−18 h.

### Transformation of chemically competent cells

To transform cells, 100 μL of competent cells were thawed on ice. To this, plasmid (2 μL each of miniprep-quality plasmid; up to two plasmids per transformation) and 100 μL KCM solution (100 mM KCl, 30 mM CaCl_2_, and 50 mM MgCl_2_ in H_2_O) were added and flicked to mix. For transformations of greater than two plasmids, 2 µL of each plasmid was added to 30 µL competent cell/KCM mix. The mixture was incubated on ice for 10 min and heat-shocked at 42 °C for 90 s. The mixture was chilled on ice for 4 min, then 1 mL of 2XYT media was added. Cells were allowed to recover at 37 °C with shaking at 230 × *g* for at least 45 min, streaked on 2XYT media + 1.5% agar plates containing the appropriate antibiotics, and incubated at 37 °C for 16−18 h.

### Selection criteria for representative E. coli tRNAs scaffolds

In cases where quadruplet-decoding tRNAs had been previously engineered, evolved, or discovered as natural suppressors, the same tRNA scaffolds were used in our evolution studies. This criterion was met for four tRNAs (Gly, Leu, Ser, Thr). In cases where either only a single copy of the tRNA scaffold was present in the *E. coli* genome (Cys, Trp) or all copies are genotypically identical with exception of the anticodon (Asn, Asp, Glu, His, Ile, Lys, fMet, Phe, Tyr), our choice was constrained to a single genotype. This criterion was met for eleven tRNAs. In all other cases, we chose tRNA scaffolds that were found at the end of their respective operons^82^ or sufficiently separated from neighboring tRNAs in order to use tRNAs that are endogenously expressed at high levels, ensure that we would not inadvertently capture >1 tRNA during amplification and cloning into the tRNA expression plasmid, and limit amplification issues using primers that may anneal within the highly structured sequence of a neighboring tRNA. This criterion was met for the remaining six tRNA (Ala, Met, Pro, Gln, Arg, Val). tRNA genes were amplified directly from *E. coli* genomic DNA.

### LacZ selections

To nominate positions in *lacZ* for amino-acid-specific selections, we first confirmed the dependence of key positions in *lacZ* on the identity of the incorporated amino acid. Oligos bearing degenerate triplet codons (NNN) at requisite positions in *lacZ* were used to generate libraries of the constitutive *lacZ*-expressing plasmids pAB191b using USER cloning. Chemically competent BW25113 ∆lacI (Strain JW0336-1; CGSC#: 8528) cells were transformed with the libraries, recovered for 2 h at 37 °C with shaking at 230 × *g*, then plated as a serial dilution series on M9 minimal medium plates with glucose 1.8% agar, 0.01% thiamine, 22 mM glucose, 40 µg/mL chloramphenicol) or lactose (1.8% agar, 0.01% thiamine, 11 mM lactose, 0.033% Bluo-Gal, 40 µg/mL chloramphenicol) and incubated at 37 °C for 24−72. Libraries all showed >100-fold coverage as gauged by transformation efficiency (>6400 total CFUs), and a comparison of the total (glucose) to LacZ + (lactose) transformants were used to inform amino acid dependence. For positions where the observed frequency agreed with the expected frequency of LacZ+ colonies, 16−32 unique colonies were picked and surveyed by Sanger sequencing at the randomized residue. Positions that showed triplet codons exclusive to a single amino acid were used for quadruplet codon-qtRNA coevolution studies. For these library-cross-library selections, the identical protocol was used to generate the *lacZ* codon libraries with the exception that the used oligos encoded fully degenerate quadruplet codons (NNNN) at the requisite positions. Following plating on glucose, transformed colonies were used to make competent cells, which were later transformed with qtRNA libraries bearing fully degenerate anticodons (NNNN). Co-transformation efficiencies corresponded to >100-fold in all cases (>6.6E6 total CFUs). Single clone sequencing at the codon (*lacZ*) and anticodon (qtRNA) showed the identical sequences in most cases for colonies picked from lactose plates.

### Measurement of quadruplet codon translation efficiency using luciferase reporter

S3489 cells were transformed with the luciferase-based activity reporter and qtRNA expression plasmids. Overnight cultures of single colonies grown in DRM media supplemented with maintenance antibiotics were diluted 500-fold into DRM media with maintenance antibiotics in a 96-well 2 mL deep well plate, with or without IPTG. The plate was sealed with a porous sealing film and grown at 37 °C with shaking at 900 × *g*. After 1 h, 175 μL of cells were transferred to a 96-well black-walled clear-bottom plate, and then 600 nm absorbance and luminescence were read using a CLARIOstar plate reader (Reader Control 5.21 R2, BMG Labtech) over the course of 8 h, during which the cultures were incubated at 37 °C. IPTG inducer concentration was 1 mM IPTG. For expression of orthogonal rRNA, the aTc inducer concentration was 100 ng/mL. GraphPad Prism (version 8) was used for plotting and data analysis, including calculation of means and standard deviations.

### Calculation of percent wild-type luciferase (η)

In order to robustly compare toxic and non-toxic qtRNAs, all luminescence values are considered at OD_600_ = 0.5 to account for differential growth rates. The percent of triplet codon translation efficiency, *η*, is calculated using the formula:

1 $$\eta =\frac{{{{{{{\rm{QuadLux}}}}}}}_{{{{{{{{\rm{qtRNA}}}}}}\; {{{{{\rm{induced}}}}}}}}}{{\mbox{-}}}{{{{{{\rm{QuadLux}}}}}}}_{{{{{{{{\rm{qtRNA}}}}}}\; {{{{{\rm{uninduced}}}}}}}}}\,}{{{{{{\rm{TriLux}}}}}}{{\mbox{-}}}{{{{{{\rm{QuadLux}}}}}}}_{{{{{{{{\rm{qtRNA}}}}}}\;{{{{{\rm{uninduced}}}}}}}}}}\times 100$$

Where TriLux is the luminescence of the positive control, a luciferase encoded entirely with triplet codons; QuadLux_qtRNA induced_ is the luminescence produced by the quadruplet codon-bearing reporter upon qtRNA expression (1 mM IPTG); QuadLux_qtRNA uninduced_ is the luminescence produced by the quadruplet codon-bearing reporter upon qtRNA expression (0 mM IPTG).

### Doubling time analyses

Colonies transformed with the appropriate wild-type tRNA, qtRNA, or a combination therein were picked and grown in DRM containing maintenance antibiotics. Following overnight growth at 37 °C with shaking at 900 × *g*, cultures were back diluted 100-fold into DRM containing maintenance antibiotics +/− IPTG. After growing for 1 h at 37 °C with shaking at 900 × *g* in a 96 deep well plate, 175 µl of each culture were transferred to a 96-well black wall, the clear bottom plate (Costar), and OD_600_ was measured every 10 min over 10 h. The doubling time of wild type and qtRNA cultures were calculated using the Growthcurver package (version 0.3.1)^83^ in R (version 4.0.3).

### Fluorescence assays

S3489 cells were transformed with the sfGFP and/or mCherry-based activity reporter and qtRNA expression plasmid(s). For assays containing two plasmids (one qtRNA expression plasmid and one reporter plasmid), colonies were picked directly into DRM media supplemented with maintenance antibiotics (with or without 1 mM IPTG inducer) and allowed to grow overnight. For assays containing greater than two plasmids, overnight cultures of single colonies grown in DRM media supplemented with maintenance antibiotics were diluted 500-fold into DRM media with maintenance antibiotics in a 96-well 2 mL deep well plate (with or without 1 mM IPTG inducer). For three plasmid assays, the concentration of each antibiotic was cut by one third (i.e., carbenicillin, 16.7 μg/mL) and for four plasmid assays, the concentration of each antibiotic was cut by one fourth (i.e., carbenicillin 12.5 µg/mL). In all cases, the deep well plates were sealed with a porous sealing film and grown at 37 °C with shaking at 230 × *g* for 24−36 h. 150 μL of cells were transferred to a 96-well black-walled clear-bottom plate, and then 600 nm absorbance and fluorescence (sfGFP: λEx = 485 nm and λEm = 510 nm; mCherry: λEx = 587 nm and λEm = 610 nm) were read at 37 °C using a Spark (Tecan) plate reader running SparkControl v2.3. GraphPad Prism (version 8) was used for plotting and data analysis.

### Calculation of percent wild-type sfGFP

After blank media subtraction, the percent of sfGFP triplet codon translation efficiency is calculated using the formula:

2 $$\% {{{{{\rm{wildtype}}}}}}\; {{{{{\rm{sfGFP}}}}}}=\frac{{{{{{{\rm{Quad}}}}}}}_{{{{{\mathrm{sfGFP}}}}}}/{{{{{{\rm{Quad}}}}}}}_{{{{{\mathrm{OD}}}}}600}\,}{{{{{{\rm{Average}}}}}}({{{{{\rm{wildtype}}}}}}_{{{{{{{\mathrm{sfGFP}}}}}}}}/{{{{{{\rm{wildtype}}}}}}}_{{{{{{{\mathrm{OD}}}}}}}600})}\times 100$$

Where wild-type refers to the positive control sfGFP containing no quadruplet codons; Quad_sfGFP_ refers to fluorescence produced by the quadruplet codon-bearing reporter upon qtRNA expression (1 mM IPTG); OD600 and sfGFP values are normalized to blank media first. Threshold calculations refer to the average of fluorescence produced by the quadruplet codon-bearing reporter upon qtRNA expression (0 mM IPTG).

### Sample preparation for quantification of qtRNA charging using mass spectrometry

Each qtRNA was co-expressed with C-terminal 6xHis-tagged *sfGFP* with the appropriate quadruplet codon replacing permissive residue Y151^56^ in S3489 cells. Bacterial cultures between 4 and 50 mL were grown for 36 h at 37 °C in DRM media containing IPTG inducer and appropriate antibiotics. Cultures were then pelleted and frozen at −80 °C for at least 1 day. Once thawed and weighed, appropriate volumes of cOmplete, EDTA-free Protease Inhibitor Cocktail (1 tablet per 50 mL extraction solution; Millipore Sigma) and B-PER Bacterial Protein Extraction Reagent (4 mL per gram pellet; Thermo Fisher Scientific) were added to the cell pellet and gently pipetted up and down till homogenous. Samples were incubated for 1 h rotating at room temperature and centrifuged at 16,000 × RCF for 20 min to separate soluble proteins (supernatant from insoluble proteins (pellet). Soluble proteins were purified using either Ni-NTA spin column (Qiagen) or His-Spin Protein Miniprep (Zymo Research) and eluted in 150 µL. Resultant purified His-tagged proteins were denatured for 5 min at 95 °C, and 22 µL sample was mixed with 7.5 µL 4x NuPAGE dye and 0.5 µL 1 M DTT. The resulting samples and Blue Prestained Protein Standard (New England Biolabs) were run on a 12% Bis-Tris PAGE gel (Invitrogen) at 200 mA, for 15 min at 90 V and then 35 min at 200 V, using 1x NuPAGE MES SDS Running Buffer (Thermo Fisher Scientific). The SDS-PAGE gel was then washed in DI H_2_O for 5 min three times, stained for 2 h in GelCode Blue Stain Reagent (Thermo Fisher Scientific), and destained in 50% methanol/water overnight. GelCode Blue stained SDS–PAGE gel lanes were then cut into ~2 mm squares, washed once more with 47.5/47.5/5 % methanol/water/acetic acid for 2 h, dehydrated with acetonitrile, and dried in a speed-vac. Reduction of disulfide bonds was then carried out by the addition of 30 μl 10 mM dithiothreitol (DTT) in 100 mM ammonium bicarbonate for 30 min. The resulting free cysteine residues were subjected to an alkylation reaction by removal of the DTT solution and the addition of 100 mM iodoacetamide in 100 mM ammonium bicarbonate for 30 min to form carbamidomethyl cysteine. These were then sequentially washed with aliquots of acetonitrile, 100 mM ammonium bicarbonate, and acetonitrile and dried in a speed-vac. The bands were enzymatically digested by the addition of 300 ng of trypsin (or chymotrypsin for arginine or lysine qtRNAs) in 50 mM ammonium bicarbonate to the dried gel pieces for 10 min on ice. Depending on the volume of acrylamide, excess ammonium bicarbonate was removed or enough was added to rehydrate the gel pieces. These were allowed to digest overnight at 37 °C with gentle shaking. The resulting peptides were extracted by the addition of 50 μL (or more if needed to produce supernatant) of 50 mM ammonium bicarbonate with gentle shaking for 10 min. The supernatant from this was collected in a 0.5 mL conical autosampler vial. Two subsequent additions of 47.5/47/5/5 acetonitrile/water/formic acid with gentle shaking for 10 min were performed with the supernatant added to the 0.5 mL autosampler vial. The organic solvent was removed, and the volumes were reduced to 15 μL using a speed vac for subsequent analyses.

### Chromatographic separations

The digestion extracts were analyzed by reverse-phase high-performance liquid chromatography (HPLC) using Waters NanoAcquity pumps and autosampler and a ThermoFisher Orbitrap Elite mass spectrometer using a nano flow configuration. A 20 mm × 180 μm column packed with 5 μm Symmetry C18 material (Waters) using a flow rate of 15 μl per min for 3 min was used to trap and wash peptides. These were then eluted onto the analytical column which was self-packed with 3.6 μm Aeris C18 material (Phenomenex) in a fritted 20 cm × 75 μm fused silica tubing pulled to a 5 μm tip. Elution was carried out with a gradient of isocratic 1% Buffer A (1% formic acid in H_2_O) for 1 min (250 nL min^−1^), followed by increasing Buffer B (1% formic acid in acetonitrile) concentrations to 15% B at 20.5 min, 27% B at 31 min and 40% B at 36 min. The column was washed with high percent B and re-equilibrated between analytical runs for a total cycle time of approximately 53 min.

### Mass spectrometry

The mass spectrometer was operated in a dependent data acquisition mode where the 10 most abundant peptides detected in the Orbitrap Elite (ThermoFisher) using full scan mode with a resolution of 240,000 were subjected to daughter ion fragmentation in the linear ion trap. A running list of parent ions was tabulated to an exclusion list to increase the number of peptides analyzed throughout the chromatographic run. The resulting fragmentation spectra were correlated against custom databases using PEAKS Studio X (Bioinformatics Solutions). To calculate the limit of detection and relative amino acid abundance, the results were matched to a library of GFP variants with each of the 20 canonical amino acids at respective residues. The abundance of each species was quantified by calculating the area under the curve of the ion chromatogram for each peptide precursor. The limit of detection was 10^4^ (arbitrary units), the lower limit for area under the curve for a peptide on this instrument.

### Phage supernatant filtration

To filter 500 μL of phage, bacteria were pelleted by centrifugation at 8,000 × RCF for 2 min in a tabletop centrifuge. The supernatant was transferred to a 0.22 μm filter column and centrifuged at 1000 × RCF for 1 min to create filtered phage flow-through. To filter 50 mL of phage supernatant, 50 mL of culture was similarly pelleted. The supernatant was applied to a Steriflip (Millipore Sigma) 0.22 μm vacuum filter unit. To filter up to 150 μL of phage in 96-well plate format, the 96-well plate of bacteria was pelleted by centrifugation at 1,000 × RCF for 10 min. 150 μL of supernatant was applied to wells of a 96-well 0.22 μm filter plate taped atop a 96-well PCR plate and centrifuged at 1000 × RCF for 1 min to create filtered phage flow-through. Phage can be stored at 4 °C in 96-well plate format covered with an aluminum sealing film. For frequently accessed phage samples, we recommend storage in 2 mL screw-cap tubes to minimize potential phage contamination generated from opening snap-caps.

### Standard phage cloning

Competent *E. coli* S3489 cells were prepared (as described) containing pJC175e, a plasmid expressing pIII under control of the phage shock promoter^84^. To clone ΔpIII M13 bacteriophage, PCR fragments were assembled using USER, as above. The annealed fragments were transformed into competent S3489-pJC175e competent cells (as described), which complement pIII deletion from the bacteriophage. Transformants were recovered in 2xYT media overnight, shaking at 230 × *g* at 37 °C. The phage supernatant from the resulting culture was filtered (as described), and plaqued (as described). Clonal plaques were expanded overnight, filtered, and Sanger sequenced.

### Phage library cloning

We do not recommend USER cloning for library creation inside of high-secondary structure tRNAs; instead, we used degenerate primers and blunt-end ligation. Primers were designed containing a NNNN degenerate anticodon. To reduce nucleotide bias during blunt end ligation assembly, the last degenerate base was designed to be at least one base away from the end of the primer. For each library, 200 μL of PCR product was used. The entirety of this PCR product was run on a gel, extracted, and purified using spin column purification. Background plasmid was digested using DpnI (New England Biolabs), and the remaining PCR product was purified again using spin columns, and ligated. The ligation product was transformed into competent *E. coli* S3489 cells containing pJC175e. Transformants were recovered in 2xYT media overnight, shaking at 230 × *g* at 37 °C. The phage supernatant from the resulting culture was filtered and plaqued.

### Manual phage plaque assays

S3489 cells were transformed with the Accessory Plasmid of interest. Overnight cultures of single colonies grown in 2xYT media supplemented with maintenance antibiotics were diluted 1,000-fold into fresh 2xYT media with maintenance antibiotics and grown at 37 °C with shaking at 230 × *g* to OD_600_ ~0.6–0.8 before use. Bacteriophage were serially diluted 100-fold (four dilutions total) in H_2_O. 100 μL of cells were added to 100 μL of each phage dilution, and to this 0.85 mL of liquid (70 °C) top agar (2xYT media + 0.6% agar) supplemented with 2% Bluo-Gal was added and mixed by pipetting up and down once. This mixture was then immediately pipetted onto one quadrant of a quartered Petri dish already containing 2 mL of solidified bottom agar (2XYT media + 1.5% agar). After solidification of the top agar, plates were incubated at 37 °C for 16–18 h.

### Phage enrichment assays

S3489 cells were transformed with the Accessory Plasmids of interest as described above. Overnight cultures of single colonies grown in 2XYT media supplemented with maintenance antibiotics were diluted 1,000-fold into DRM media with maintenance antibiotics and grown at 37 °C with shaking at 230 × *g* to OD_600_ ~0.4–0.6. Cells were then infected with bacteriophage at a starting titer of 10^5^ pfu/mL. Cells were incubated for another 16–18 h at 37 °C with shaking at 230 × *g*. Supernatant was filtered and stored at 4 °C. The phage titer of these samples was measured in an activity-independent manner using a plaque assay containing *E. coli* bearing pJC175e.

### Continuous flow PACE

Unless otherwise noted, PACE apparatus, including host cell strains, lagoons, chemostats, and media, were all used as previously described^66^. Chemically competent S3489 cells were transformed with the Accessory Plasmid and the mutagenesis plasmid (MP) MP6^35^ as described above, plated on 2xYT media + 1.5% agar supplemented with 25 mM glucose (to prevent induction of mutagenesis) in addition to maintenance antibiotics, and grown at 37 °C for 18–20 h. To validate MP functionality prior to evolution, four colonies were picked into 10 μL DRM media and diluted 10-fold six times; these dilutions were plated on either agar plates with maintenance antibiotics and 25 mM glucose, or 10 mM arabinose; we expected that colonies plated on arabinose would be of reduced size when the MP is functional. The remainder of the dilutions were added to 2 mL DRM media with maintenance antibiotics, grown at 37 °C with shaking until they reached OD_600_ ~0.4–0.8, and then used to inoculate a turbidostat containing 30 mL DRM media. The turbidostat maintained the growing culture at OD_600_ ~0.7–0.8. Prior to bacteriophage infection, lagoons were continuously diluted with culture from the turbidostat at 1 lagoon vol/h and pre-induced with 10 mM arabinose for at least 45 min to induce mutagenesis. Samples (500 μL) of the SP population were taken at indicated times from lagoon waste lines. These were centrifuged at 8,000 RCF for 2 min, and the supernatant was passed through a 0.22 μm filter and stored at 4 °C. Lagoon titers were determined by plaque assays using S3489 cells transformed with pJC175e.

### Aminoacyl-tRNA synthetase expression and purification

*E. coli* SerRS, ArgRS, and TyrRS were overexpressed in BL21 (DE3) *E. coli* cells and purified as previously described^35^ with slight modifications. Cells were grown at 37 °C until OD_600_ 0.6 and induced with 0.5 mM isopropyl β-D-1-thiogalactopyranoside for 3 h at 30 °C. Cells were resuspended in Buffer A (50 mM HEPES-KOH [pH 7.5], 300 mM NaCl, 10 mM β-mercaptoethanol, 3 mM MgCl_2_, 10 mM imidazole) along with a protease inhibitor tablet (Roche, cOmplete Mini, EDTA-free) and subjected to sonication. The lysate was centrifuged at 38,000 × *g* for 40 min at 4 °C and the synthetases were purified via nickel affinity chromatography. The synthetases were eluted with Buffer B (50 mM HEPES-KOH [pH 7.5], 300 mM NaCl, 10 mM β-mercaptoethanol, 3 mM MgCl_2_, 250 mM imidazole) and incubated with His-tagged TEV protease for 1 h at 37 °C. The aaRS-TEV protease solution was dialyzed into Buffer A, subjected to nickel affinity chromatography to isolate the aaRS, dialyzed into a storage buffer (50 mM HEPES-KOH [pH 7.5], 100 mM NaCl, 10 mM β-mercaptoethanol, 3 mM MgCl_2_, 50% glycerol), and stored at −80 °C.

### In vitro aminoacylation assay

All qtRNAs were in vitro transcribed using T7 RNA polymerase and gel purified as previously described^35^. Prior to use, the qtRNAs were heated to 85 °C and slowly cooled down to room temperature in the presence of 10 mM MgCl_2_ to allow proper refolding. In vitro aminoacylation of tRNA^Ser^_UAGA_ by *E. coli* seryl-tRNA synthetase, tRNA^Arg^_UAGA_ by *E. coli* arginyl-tRNA synthetase, and tRNA^Tyr^_UAGA_ by *E. coli* tyrosyl-tRNA synthetase were performed as previously described^65^. Briefly, reactions contained 50 mM HEPES-KOH [pH 7.3], 4 mM ATP, 25 mM MgCl_2_, 0.1 mg/mL bovine serum albumin, 20 mM KCl, 20 mM 2-mercaptoethanol, 4 µM qtRNA, amino acid (25 µM L-[^14^C]-Ser, 6 µM L-Arg (2 µM L-[^14^C]-Arg, 4 µM L-Arg), or 6 µM L-Tyr (2 µM L-[^14^C]-Tyr, 4 µM L-Tyr)) and *E. coli* aaRS (50 mM SerRS, 30 nM ArgRS, or 30 nM TyrRS). The reactions were incubated at 37 °C and 8 µL aliquots were removed at given intervals, spotted onto 3 MM filter papers (presoaked with 5% trichloroacetic acid and dried), immersed in 5% TCA to precipitate aminoacyl-qtRNAs, and then subjected to scintillation counting.

### Diagrams and Crystal Structures

R2R (version 1.0.6) was used to generate tRNA diagrams. R2R is free software available from https://sourceforge.net/projects/weinberg-r2r/. The co-crystal structure of *E. coli* tRNA-Gln and glutaminyl-tRNA synthetase was visualized in PyMOL version 2.5.0.

### Reporting summary

Further information on research design is available in the Nature Research Reporting Summary linked to this article.

## Supplementary information

Supporting Information

Reporting Summary

## Data Availability

The data found in figures: 1b−e, 2a, d−g, 3a−c, 4a, b, d, 5a, d−f, h−k, and supplementary figures: 1, 2a, b, 5, 8, 9, 12, and 13 are available in the associated Source Data File. Select plasmids have been deposited to Addgene (deposit ID 77424, material ID #134787-134808, and 134812-134814) and uploaded to Benchling (see Extended Supplement for links). The remaining plasmid maps will be provided upon request. Source data are provided with this paper.

## Source data

Source Data
