# Supplementary material for: Multiplex suppression of four quadruplet codons via tRNA directed evolution
Source: Nat Commun. 2021 Sep 29;12:5706. doi: 10.1038/s41467-021-25948-y (PMC8481270; doi:10.1038/s41467-021-25948-y)
Supplement: Supplementary file 1 — Supporting Information [file 41467_2021_25948_MOESM1_ESM.docx]

*SUPPORTING INFORMATION*

***Multiplex Suppression of Four Quadruplet Codons via tRNA Directed Evolution***

Erika A. DeBenedictis^1,2*^, Gavriela D. Carver^1*^, Christina Z. Chung^3^, Dieter Söll^3,4^, Ahmed H. Badran^1,5**^

^1^ The Broad Institute of MIT & Harvard, Cambridge MA 02142

^2^ Department of Biological Engineering, Massachusetts Institute of Technology, Cambridge MA 02142

^3^ Department of Molecular Biophysics and Biochemistry, Yale University, New Haven CT 06511

^4^ Department of Chemistry, Yale University, New Haven CT 06520

^5^ Department of Chemistry, The Scripps Research Institute, La Jolla, CA 92037

* These authors contributed equally: Erika A. DeBenedictis, Gavriela D. Carver

** Correspondence should be addressed to Ahmed H. Badran: [ahbadran@broadinstitute.org](mailto:ahbadran@broadinstitute.org)

**Keywords: Frameshift suppressor, quadruplet codons, genetic code expansion.**

EAD: 0000-0002-7933-2651

GDC: 0000-0001-9371-4157

CZC: 0000-0001-7761-8165

DS: 0000-0002-3077-8986

AHB: 0000-0002-8105-1883


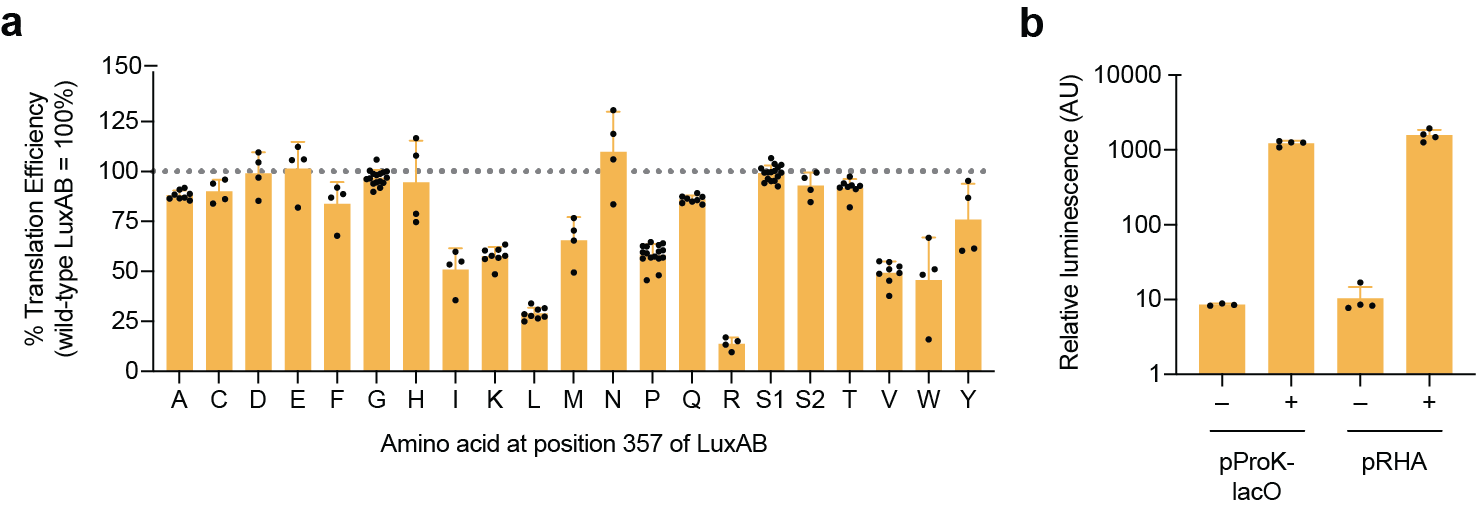


**Supplementary Figure 1 | Validation of LuxAB reporter and engineered qtRNAs.** **a**) Constitutive LuxAB reporters bearing all twenty canonical amino acids show limited preference at positive S357, with the exception of arginine which shows a five-fold reduction in luminescence activity. S1 corresponds to the UCG serine codon and S2 corresponds to the ACG serine codon at position S357. (n = 4 biologically independent samples except for A, K, L, Q, T and V n = 8, as well as G, P and S1 n = 16.) **b**) Comparison of the engineered pProk-lacO promoter to the rhamnose operon-derived pRHA promoter. In all cases, reporter data is normalized to an otherwise wild-type protein. (n = 4 biologically independent samples except for pProK-lacO (-) n = 3.) Data represents the mean and standard deviation as appropriate. AU: arbitrary units.


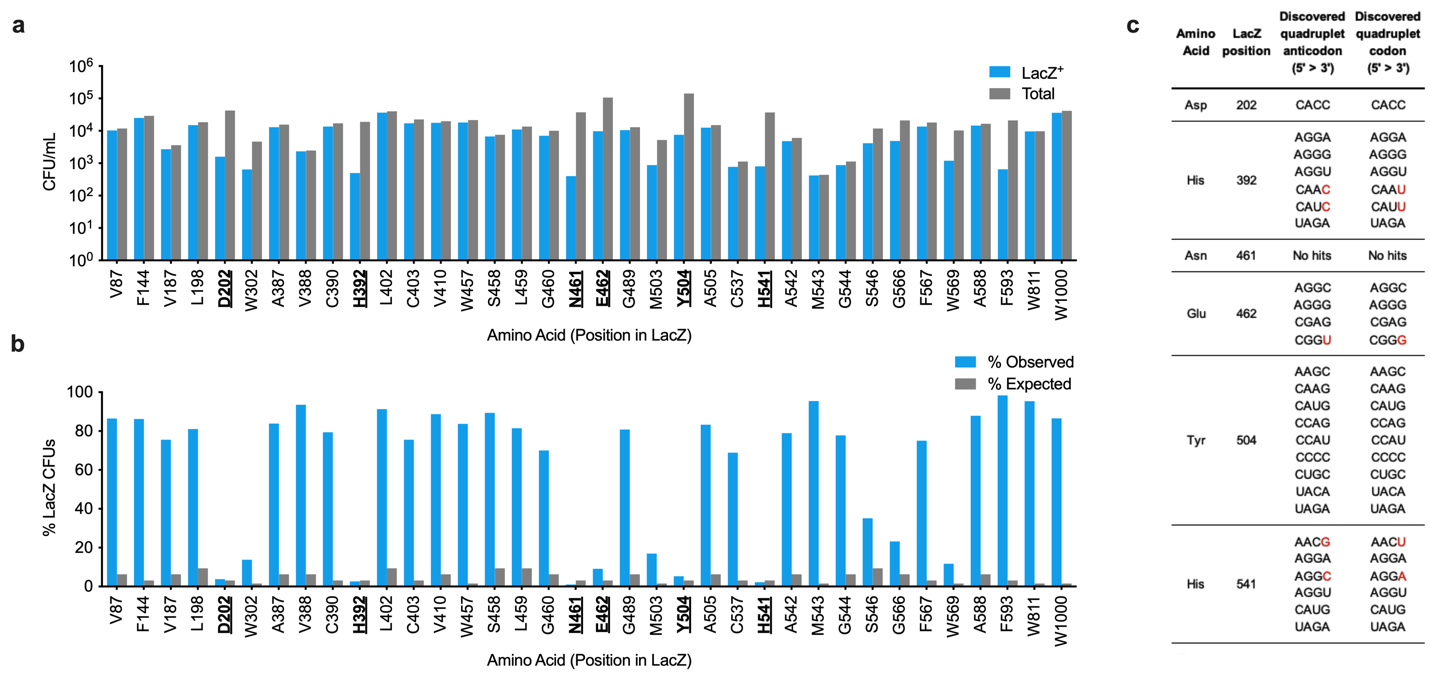


**Supplementary Figure 2 | Validation of LacZ library-cross-library selection and discovered hits. a**) To ensure that qtRNAs were discovered in an amino acid-specific manner, we first nominated positions within the *lacZ* gene for functional selections. Functional *lacZ* genes can be easily selected via plating on lactose. Degenerate (NNN) codon libraries were first incorporated in *lacZ* at all the indicated positions and plated on minimal medium plates with either glucose (“Total”) or lactose + Bluo-Gal (“LacZ^+^”). Functional amino acid incorporation results in growth on minimal media plates supplemented with lactose as the sole carbon source, and Bluo-Gal was added to confirm that colony formation was indeed dependent on LacZ. If the sizes of the total library and the lactose-catabolizing population are similar, then the position under investigation was deemed non-specific to a given amino acid. However, if the ratio of LacZ+ to total cells was <1, then this indicated that only a subset of the library led to a functional *lacZ* gene. This result would indicate that this position may be amino acid specific. **b**) Comparison of glucose- and lactose-derived populations can be used to calculate the % LacZ (% LacZ = LacZ^+^/Total *100) and the % expected LacZ^+^ CFUs assuming complete coverage of all 64 triplet codons. If the position under investigation is likely to be amino acid-specific, then we would expect both values to be comparable. In cases where both values are comparable (underlined and bold), single clone Sanger sequencing confirmed that only the cognate amino acid was present in all blue (lactose catabolizing) colonies. **c**) Amino acid-specific positions were used as the basis of a library-cross-library selection, wherein each *lacZ* position was randomized to all possible quadruplet codons (NNNN) and each tRNA scaffold was concomitantly randomized at the anticodon loop (NNNN). Co-transformation of both libraries resulted in colony growth on minimal medium plates supplemented with lactose + Bluo-Gal in all cases except N461. Single clone sequencing at the codon (*lacZ*) and anticodon (qtRNA) showed the identical sequences in most cases. The reported sequences were discovered as anticodons (reverse complement), where red letters indicate mismatches found in the *lacZ* codon. CFU: colony forming unit.

**
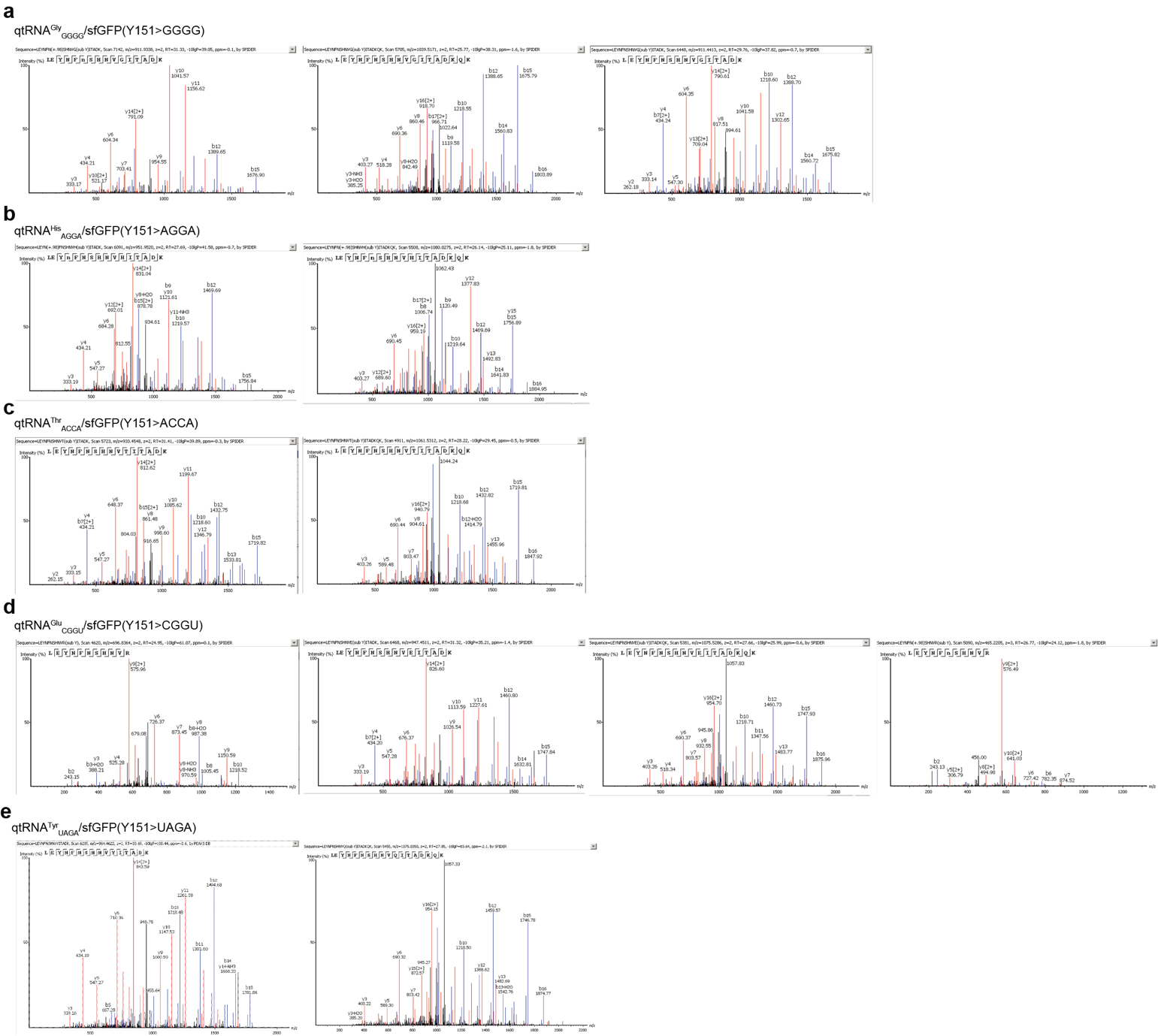
**

**Supplementary Figure 3 | LC-MS/MS analysis of lacZ selection-derived hits.** Mass spectra of sfGFP fragments resulting from qtRNA^Gly^_GGGG_ (**a**), qtRNA^His^_AGGA_ (**b**), qtRNA^Thr^_ACCA_ (**c**), qtRNA^Glu^_CGGU_ (**d**), and qtRNA^Tyr^_UAGA_ (**e**) suppression of cognate quadruplet codon at Y151. Multiple peptides were observed in some cases and are shown for completeness. Summary LC-MS/MS results are reported in **Supplementary Table 8**.

**
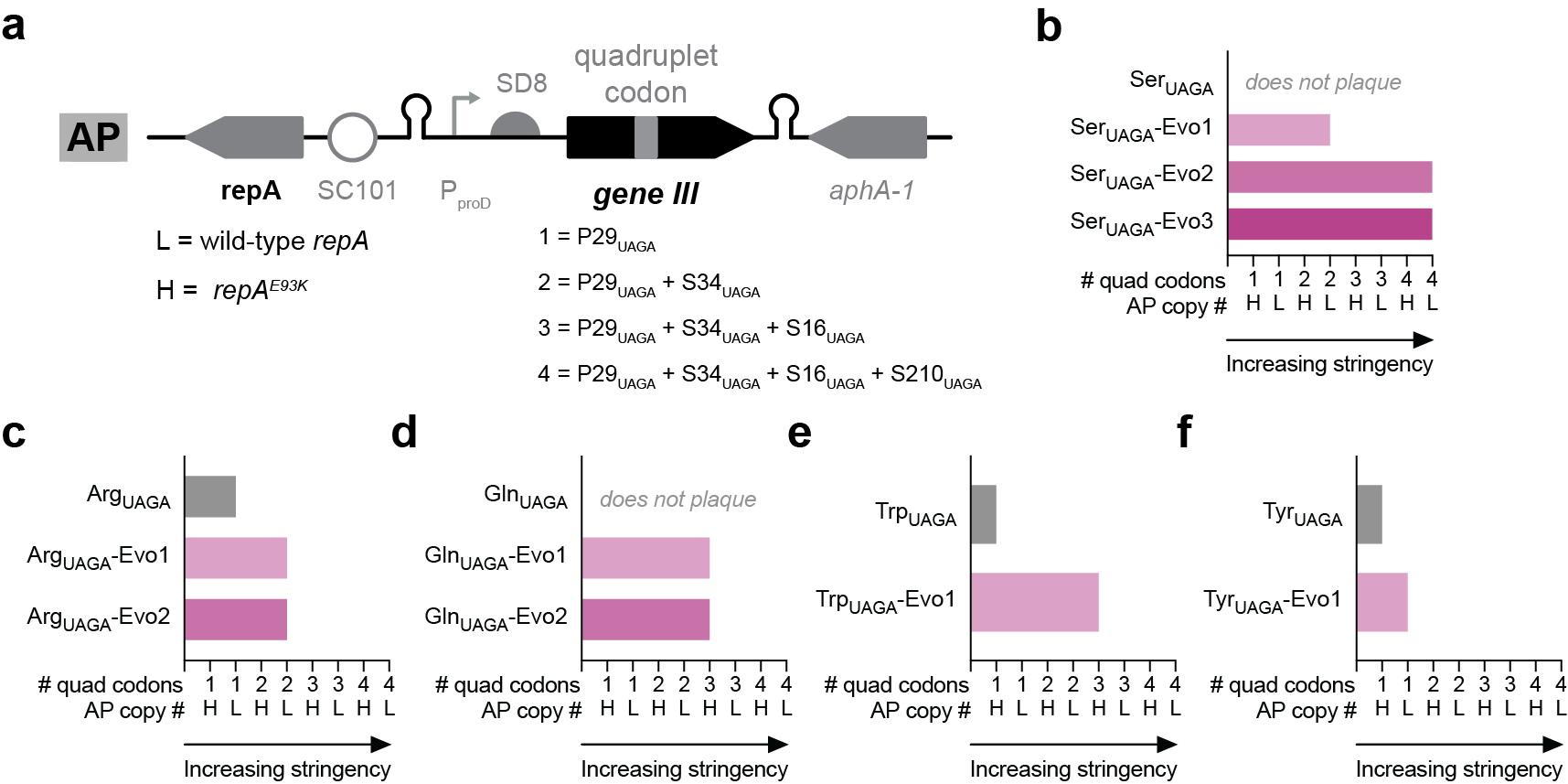
**

**Supplementary Figure 4 | Benchmarking PACE-evolved qtRNA SPs using progressively stringent APs.** **a**) Schematic representation of the accessory plasmid design, wherein either AP copy number was modified (L = wild-type *RepA* ~4 copies/cell; H = *RepA^E93K^* ~27 copies/cell) or the number of quadruplet codons in pIII was progressively increased. In all cases, clonal SPs encoding the indicated engineered or evolved qtRNAs were challenged to form plaques in S3489 cells. For each SP, the threshold for plaque formation is visualized for serine (**b**), arginine (**c**), glutamine (**d**), tryptophan (**e**), and tyrosine (**f**).


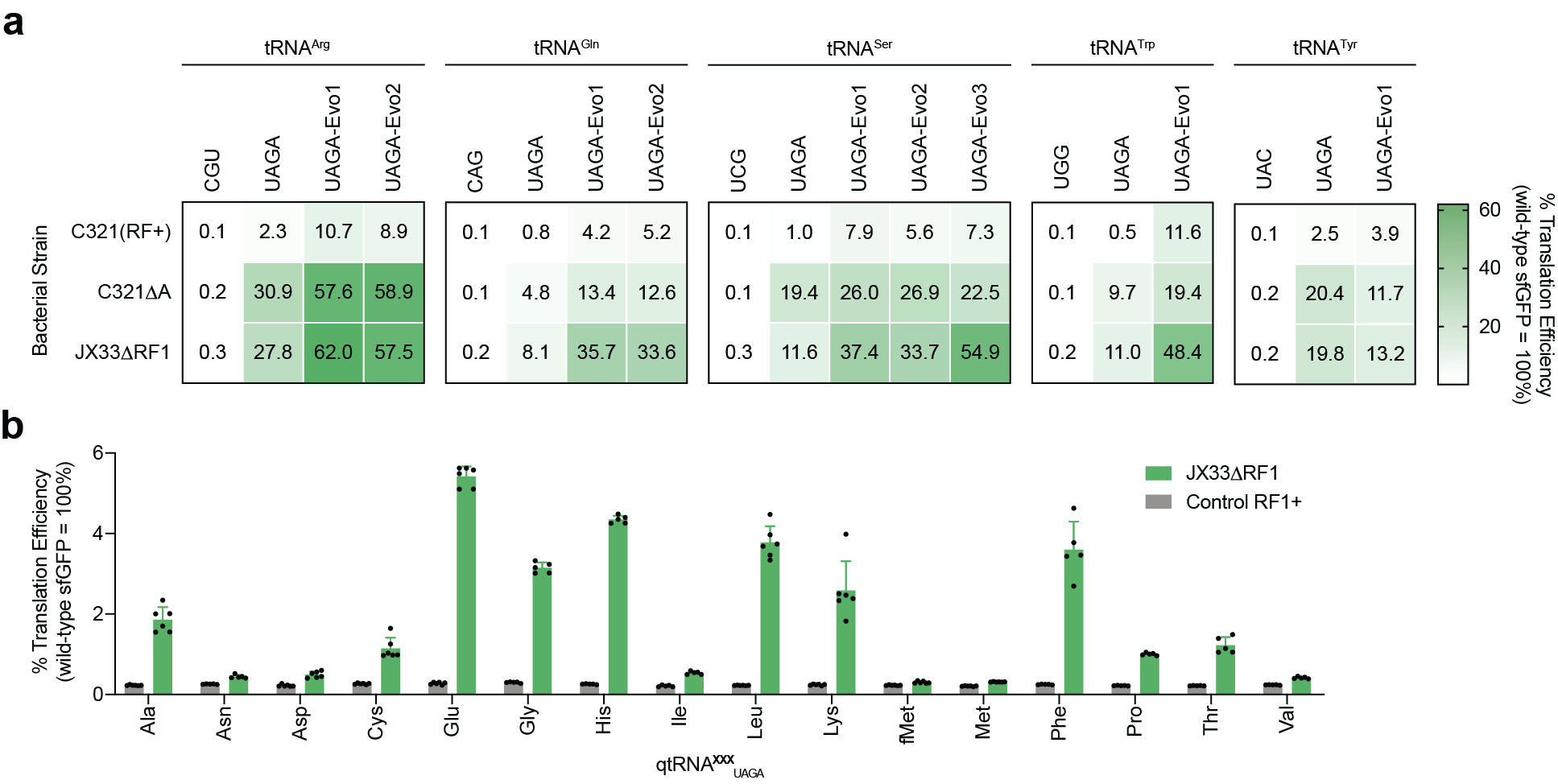


**Supplementary Figure 5 | Analysis of engineered and evolved qtRNAs in bacterial RF1 knockout strains. a**) Engineered and evolved UAGA-decoding qtRNAs assayed using an endpoint fluorescence reporter assay using two RF1 knockout strains (C321.ΔA^1^ and JX33^2^) with one RF1+ strains (C321). In all cases, tRNAs were assayed alongside a reporter incorporating the quadruplet codon UAGA at sfGFP position Y151. **b**) Extension of the sfGFP reporter assay in JX33 and S3489 (control RF+) to all rationally engineered UAGA-decoding qtRNAs (n = 6 biologically independent samples except for Asn, Gly, His, Ile, Phe, Pro, Thr, and Val where n = 5). In all cases, reporter data is normalized to an otherwise wild-type protein. Data represents the mean and standard deviation as appropriate.


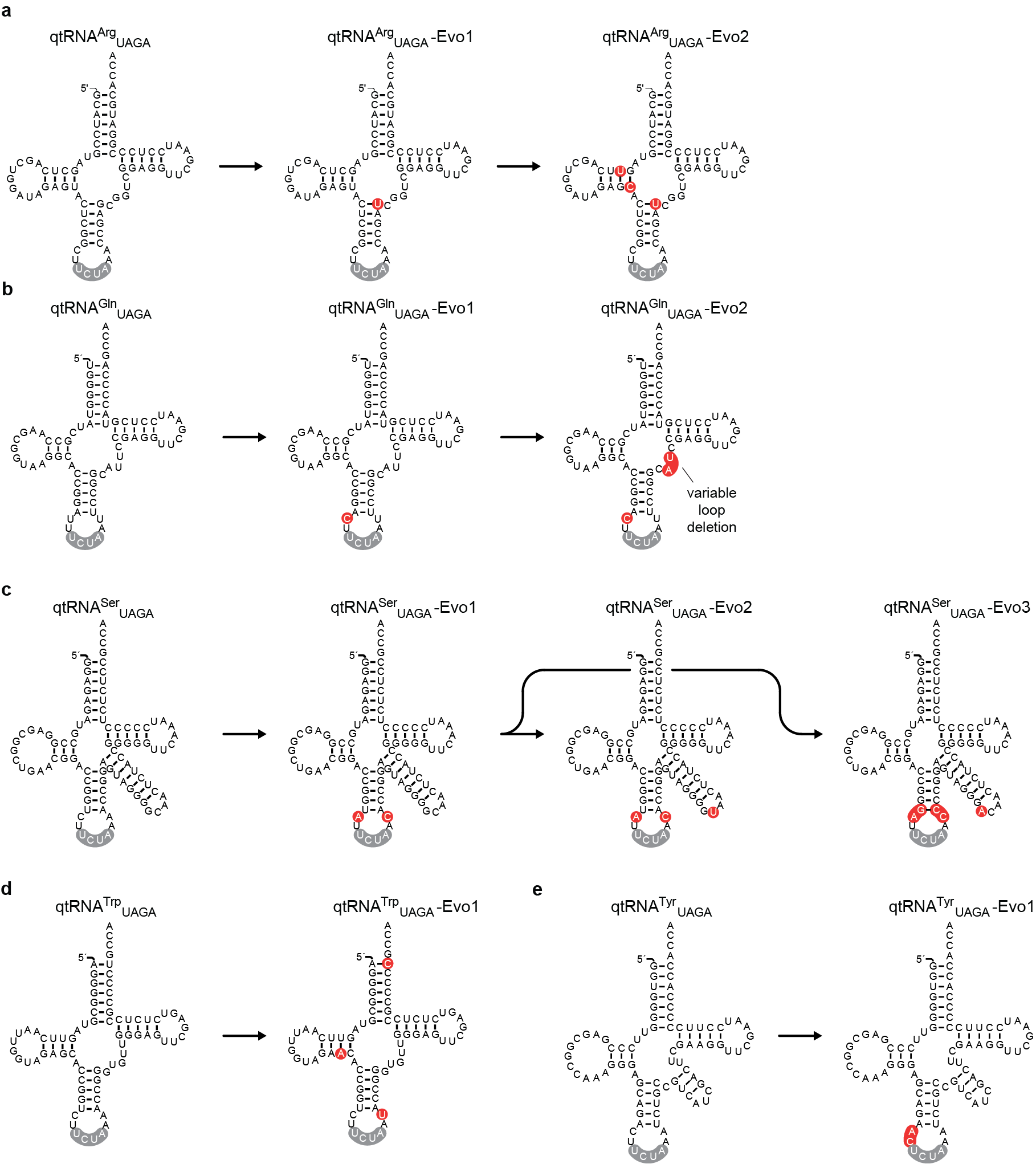


**Supplementary Figure 6 | Models of engineered and evolved qtRNAs.** Cloverleaf models of engineered UAGA qtRNAs and evolved variants: arginine (**a**), glutamine (**b**), serine (**c**), tryptophan (**d**), and tyrosine (**e**). In all cases, the engineered UAGA codon is highlighted in gray, and PACE-acquired mutations are highlighted in red. qtRNA^Ser^_UAGA_-Evo1 was used to initiate the experiment that produced qtRNA^Ser^_UAGA_-Evo2 and qtRNA^Ser^_UAGA_-Evo3.


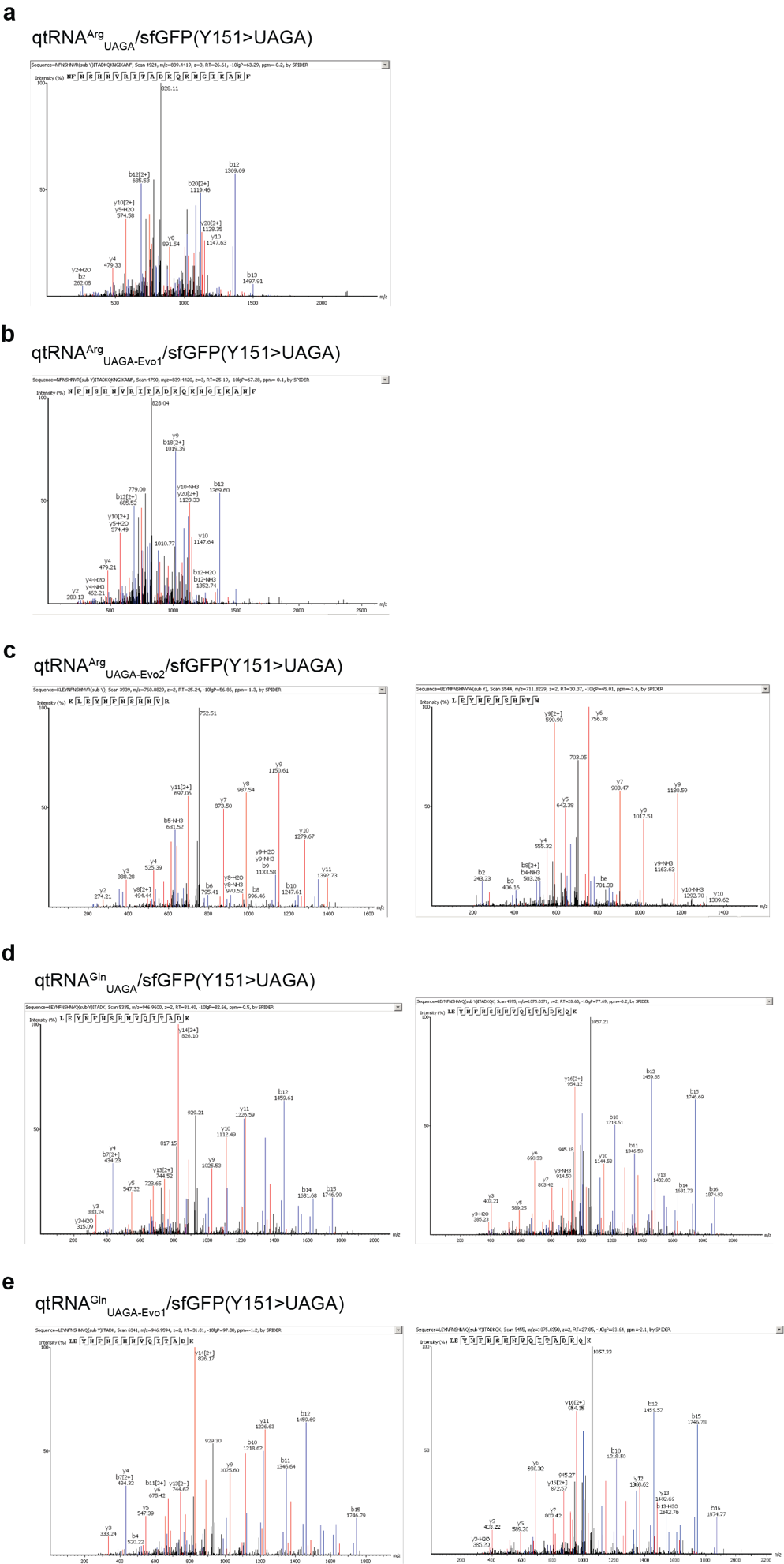


**
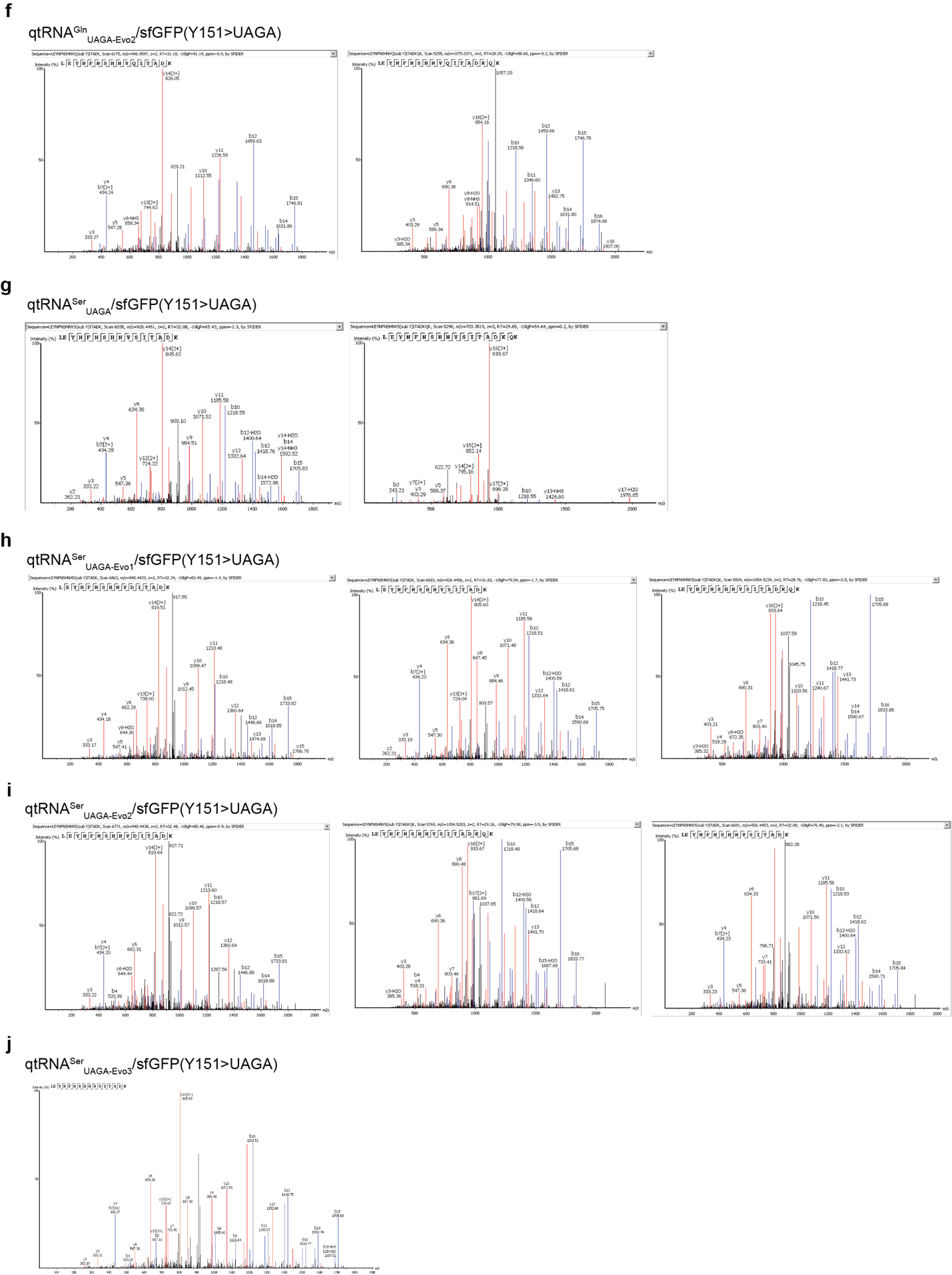
**

**
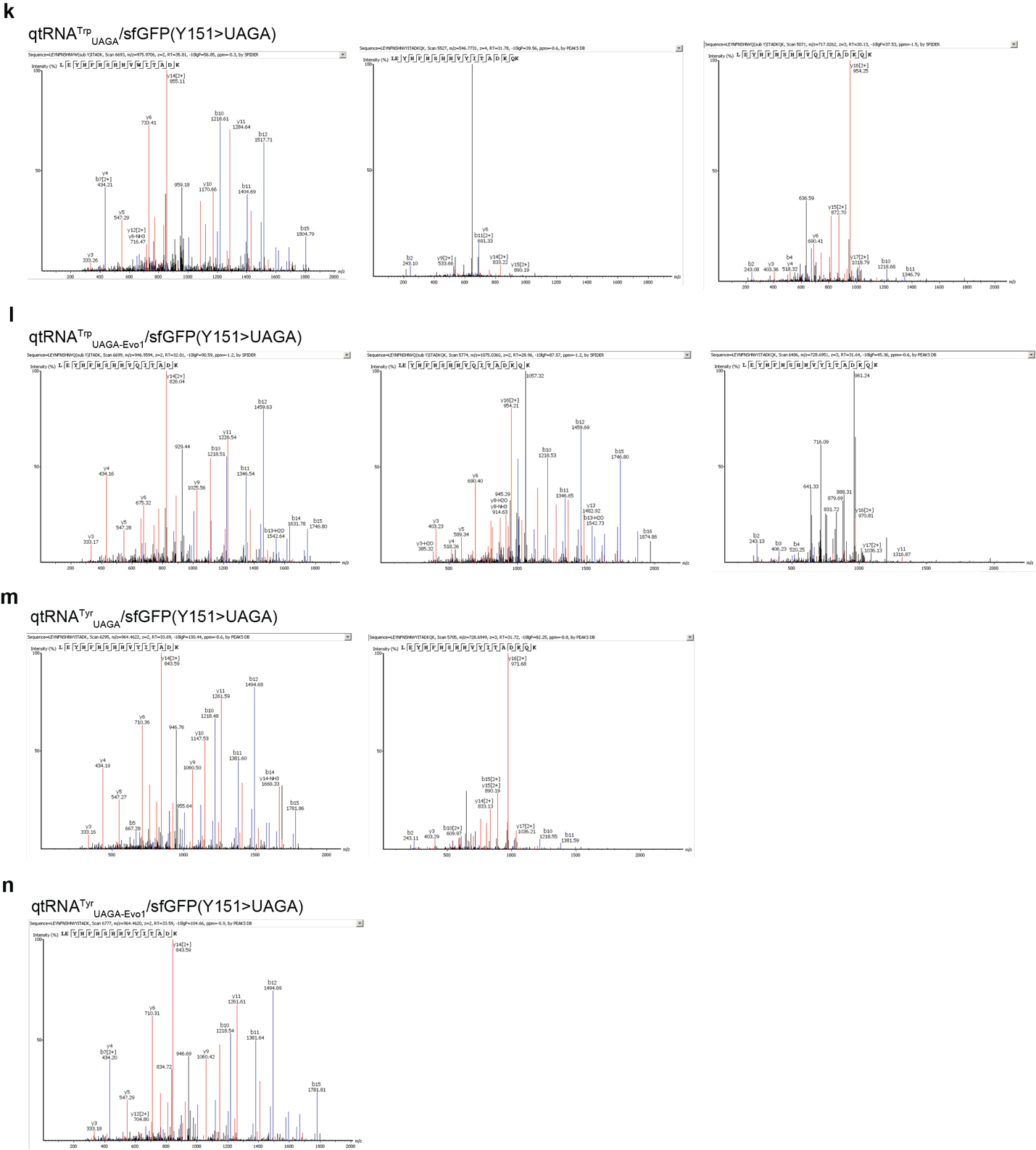
**

**Supplementary Figure 7 | LC-MS/MS analysis of engineered and evolved qtRNAs.** Mass spectra of the resultant sfGFP fragments from the suppression of UAGA quadruplet codon at sfGFP Y151 by the engineered and subsequently evolved qtRNAs: qtRNA^Arg^_UAGA_ (**a-c**), qtRNA^Gln^_UAGA_ (**d-f**), qtRNA^Ser^_UAGA_ (**g-j**), qtRNA^Trp^_UAGA_ (**k,l**), and qtRNA^Tyr^_UAGA_ (**m,n**). Multiple peptides were observed in some cases and are shown for completeness. Summary LC-MS/MS results are reported in **Supplementary Table 8**.

**
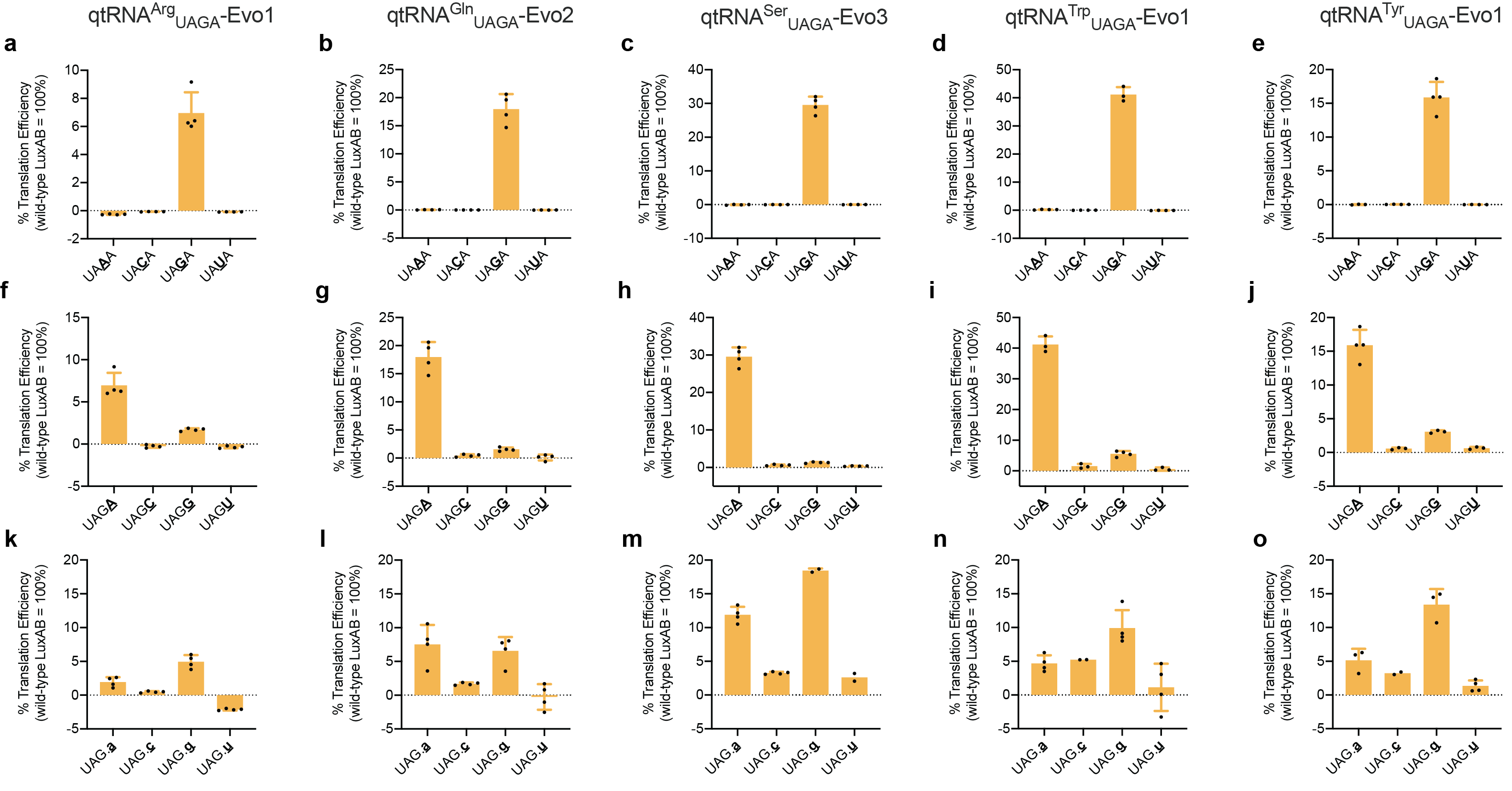
**

**Supplementary Figure 8 | Analysis of qtRNA/codon specificity and crosstalk.** Evolved UAGA-qtRNAs were tested using mismatched codon reporters to assess instances of decoding crosstalk. LuxAB reporters encoding quadruplet codons with modifications at the third position (**a-e**) or fourth position (**f-j**) showcase absolute requirement for guanine at the third position and preference for adenine at the fourth position. **k-o**) Evolved UAGA-qtRNAs continue to crosstalk with amber (UAG) stop codons, with a moderate preference for purines at the first position of the subsequent codon. In all cases, LuxAB reporter data is normalized to an otherwise wild-type protein. Data represents the mean and standard deviation of 4 biologically independent samples except for Trp-UAGA-Evo1 UA**G**A/UAG**A**/UAG**C**/UAG**U** and Tyr-UAGA-Evo1 UA**A**A/UAG**C**/UAG**G**/UAG**U**/UAG.**a**/UAG.**g** where n = 3 as well as Ser-UAGA-Evo3 UAG.**g**/UAG.**u**, Trp-UAGA-Evo1 UAG.**c**, and Tyr-UAGA-Evo1 UAG.**c** where n = 2).

**
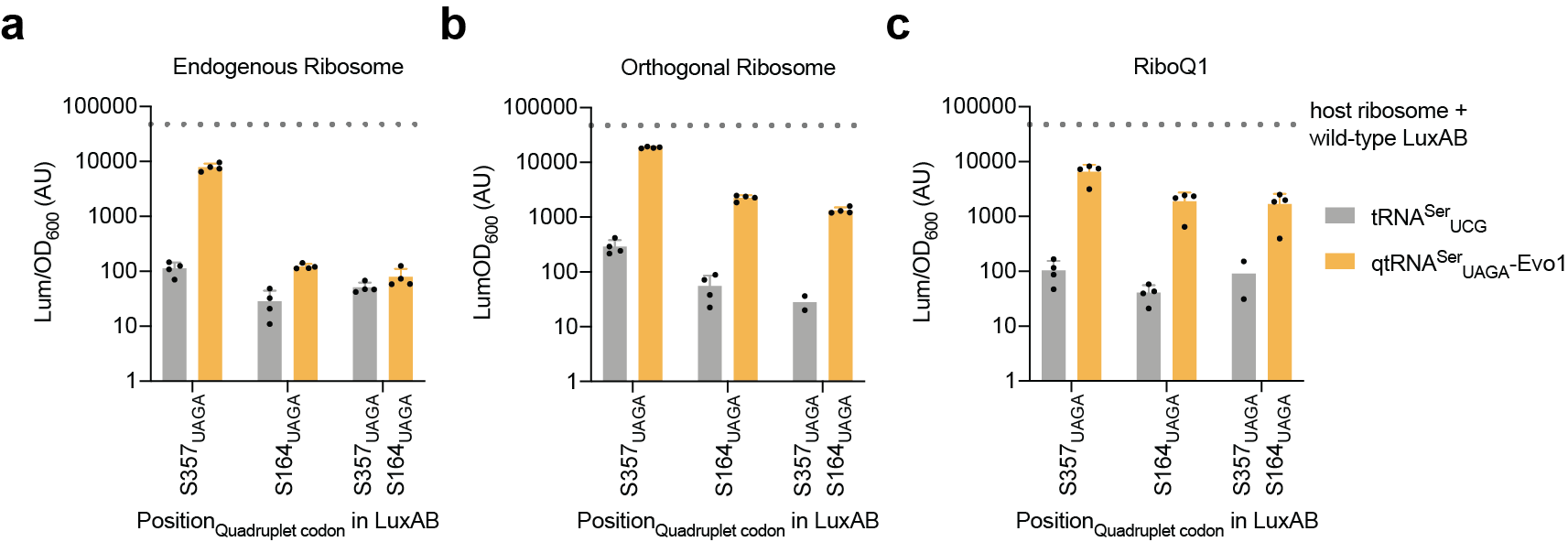
**

**Supplementary Figure 9 | Translation using orthogonal ribosome.** **a)** Translation of a reporter containing a UAGA codon at either residue 357 or residue 164, in comparison to translation of a luciferase containing UAGA codons at both locations (n = 4 biologically independent samples). **b)** Using the H3 o-RBS/o-antiRBS pair (5’-AUAUGU/5’-AUGUUC), qtRNA^Ser^_UAGA_-Evo1 translates UAGA quadruplet codons at both S357 and S164 more efficiently than when using the host ribosome, especially for reporters with multiple frameshifts (n = 4 biologically independent samples except for S357/S164+tRNA-Ser-UCG where n = 2). **c)** Orthogonal ribosomes incorporating the described RiboQ1 mutations (U531G/U534A/A1196G/A1197G)^3^ show comparable luminescence to the host wildtype ribosome for quadruplet codon translation (n = 4 biologically independent samples except for S357/S164+tRNA-Ser-UCG where n = 2). In all cases, the average wild-type (triplet) LuxAB reporter activity is shown as a dashed line. Data represent the mean and standard deviation as appropriate. OD optical density, AU arbitrary units.

**
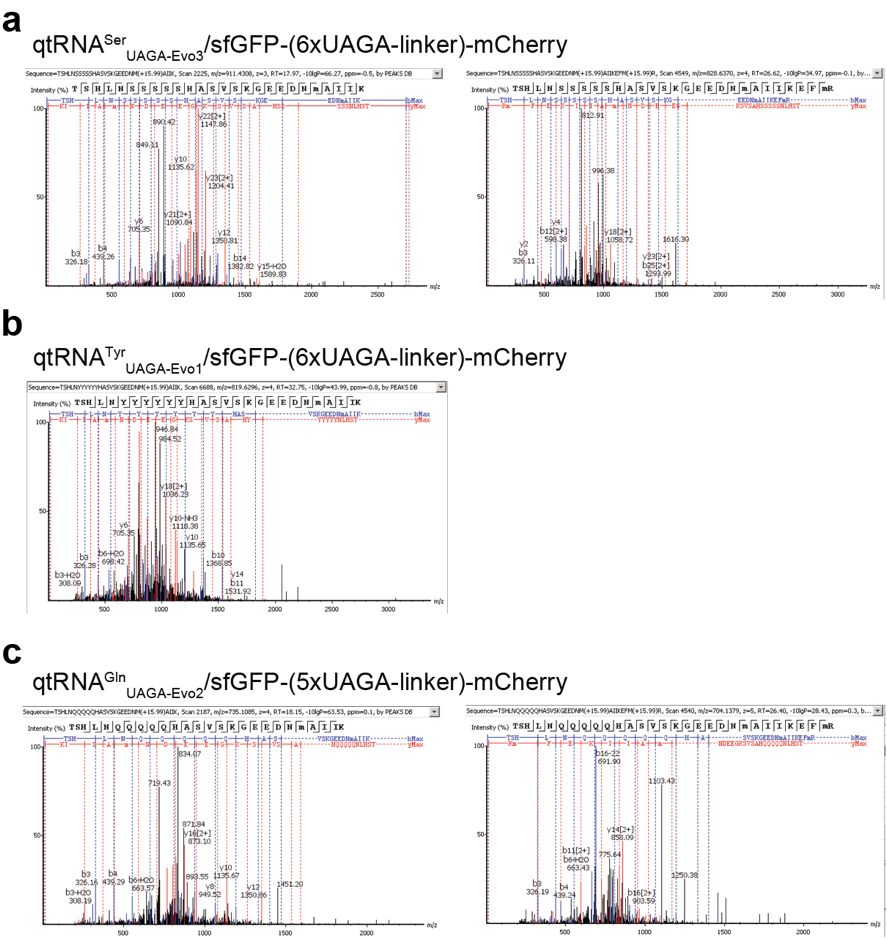
**

**Supplementary Figure 10 |** **LC-MS/MS analysis of evolved qtRNA translating a linker containing adjacent UAGA quadruplet codons.** Mass spectra of sfGFP-linked-mCherry fragments resulting from qtRNA^Ser^_UAGA_-Evo3 (**a**) and qtRNA^Tyr^_UAGA_-Evo1 (**b**) suppression of a linker containing six adjacent UAGA quadruplet codons, and qtRNA^Gln^_UAGA_-Evo2 (**c**) suppression of a linker containing five adjacent UAGA quadruplet codons. Mass spectra of the linker fragment resulting from qtRNA^Arg^_UAGA_-Evo1 and qtRNA^Trp^_UAGA_-Evo1 were unable to be identified, likely due to peptide hydrophobicity limiting chromatographic separation. Multiple peptides were observed in some cases and are shown for completeness. Summary LC-MS/MS results are reported in **Supplementary Table 8**.

**Supplementary Figure 11 |** **LC-MS/MS analysis of qtRNA translating cognate quadruplet codons at positions throughout sfGFP.** Mass spectra of sfGFP fragments resulting from qtRNA^His^_AGGA_ suppression of its cognate quadruplet codon at H148 (**a**), qtRNA^Gly^_GGGG_ suppression of its cognate quadruplet codon at G174 (**b**), qtRNA^Ser^_UAGA_-Evo3 suppression of its cognate quadruplet codon at S202 (**c**), and qtRNA^Glu^_CGGU_ suppression of its cognate quadruplet codon at E213 (**d**). Multiple peptides were observed in some cases and are shown for completeness. Summary LC-MS/MS results are reported in **Supplementary Table 8**.

**
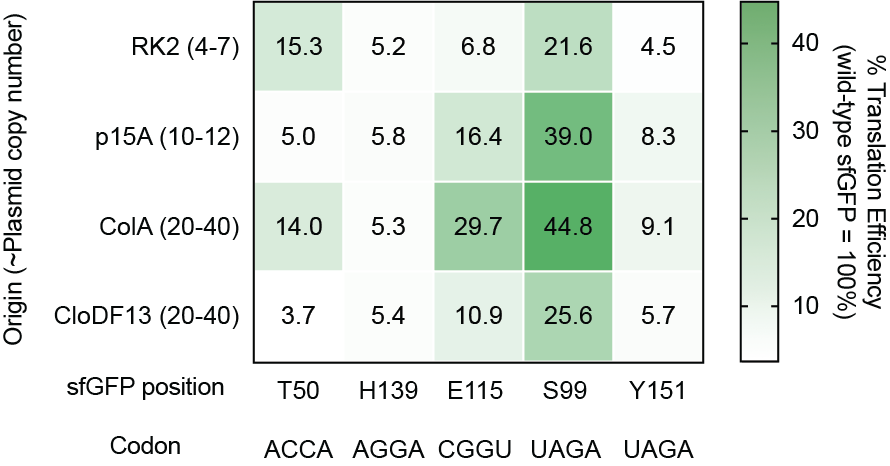
**

**Supplementary Figure 12 | Influence of plasmid copy number on qtRNA decoding efficiencies.**

qtRNAs were tested alongside cognate quadruplet codons at positions in sfGFP to assess optimal plasmid copy number (in parentheses). In all cases, reporter data is normalized to an otherwise wild-type protein. Data represents the mean and standard deviation of 8 biological replicates.

**
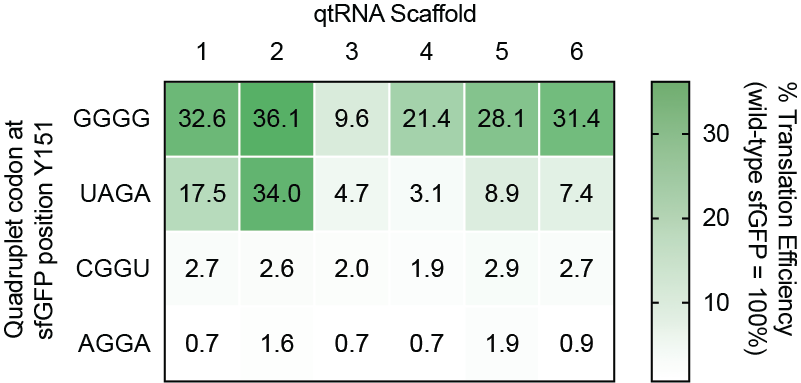
**

**Supplementary Figure 13 | Quantification of multicistronic qtRNA scaffold-based suppression.** All qtRNA scaffolds were assayed against quadruplet codons introduced at position Y151 of sfGFP. In all cases, reporter data is normalized to an otherwise wild-type protein. Data represents the mean and standard deviation of 5 biological replicates.

**
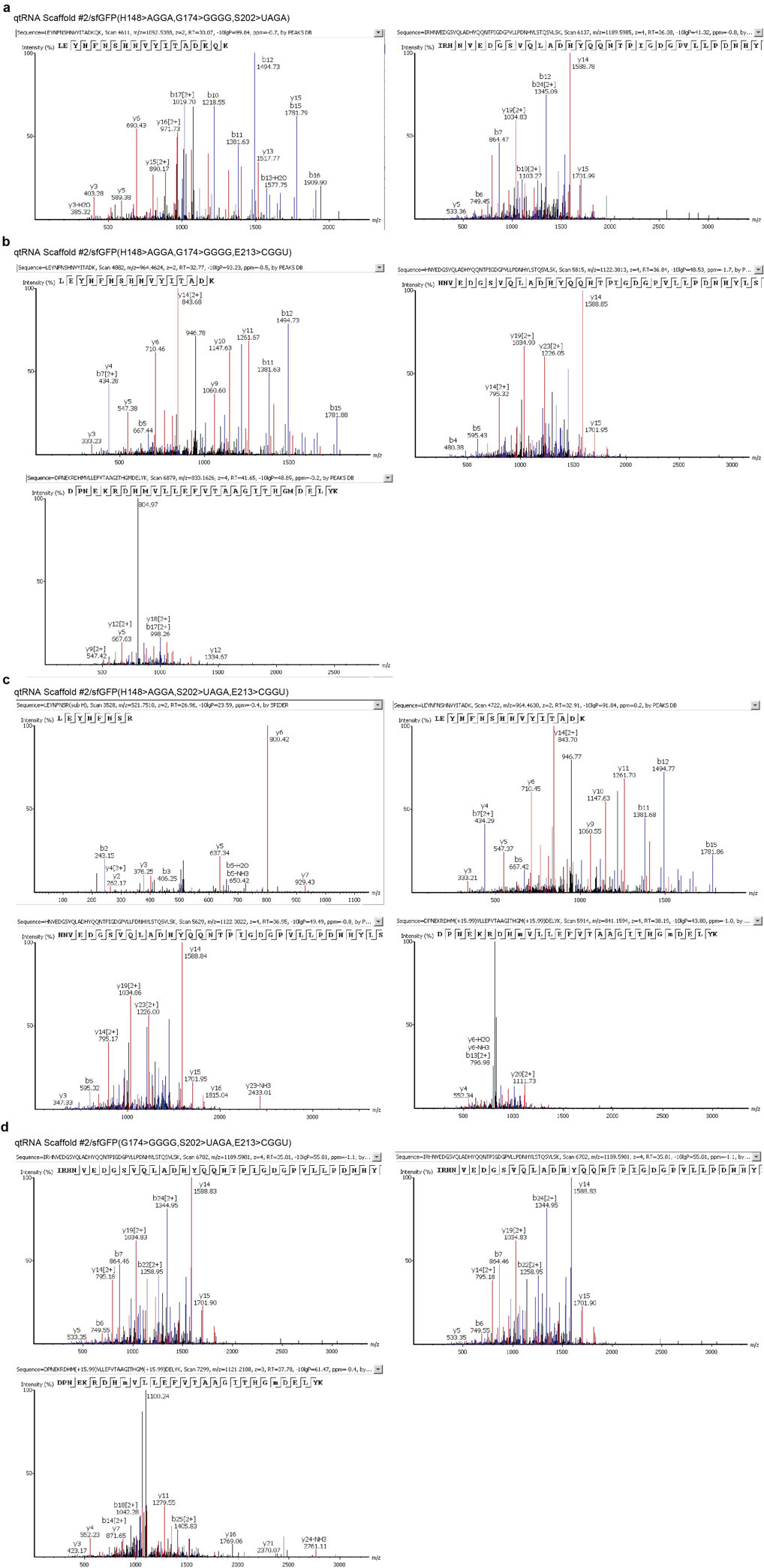
**

**Supplementary Figure 14 | LC-MS/MS analysis of qtRNA scaffold translating quadruplet codons at positions throughout sfGFP.** Mass spectra of sfGFP fragments resulting from qtRNA scaffold #2 (composed of qtRNA^Gly^_GGGG_, qtRNA^Ser^_UAGA_-Evo3, qtRNA^Glu^_CGGU_, and qtRNA^His^_AGGA_ stitched together) suppression of cognate quadruplet codons at H148, G174, and S202 (**a**), H148, G174, and E213 (**b**), H148, S202, and E213 (**c**), and G174, S202, and E213 (**d**). Multiple peptides were observed in some cases and are shown for completeness. Summary LC-MS/MS results are reported in **Supplementary Table 8**.

**
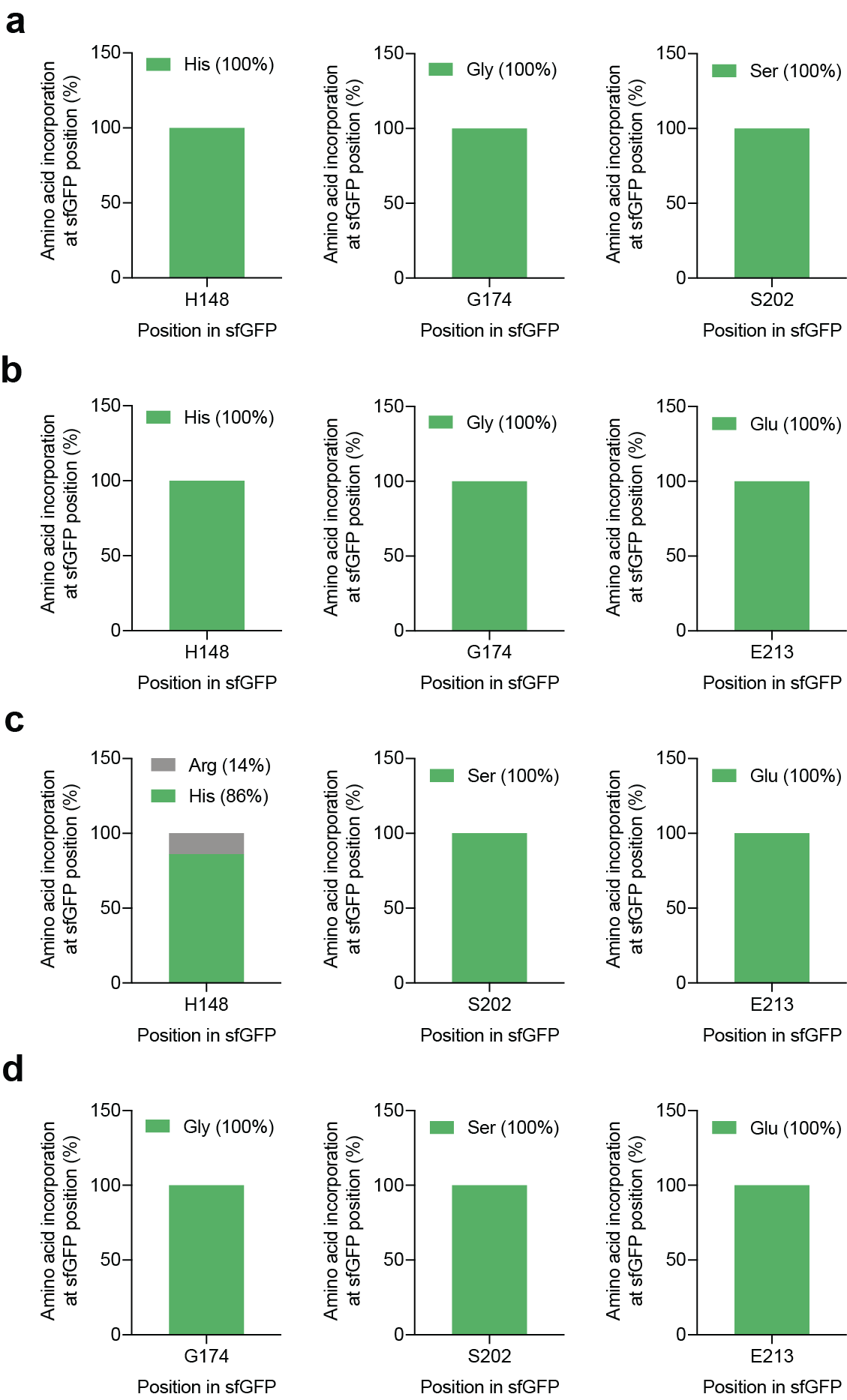
**

**Supplementary Figure 15 | Amino acid incorporation analysis corresponding to translation of three quadruplet codons in sfGFP**. Amino acid composition analysis of qtRNA scaffold #2 (composed of qtRNA^Gly^_GGGG_, qtRNA^Ser^_UAGA_-Evo3, qtRNA^Glu^_CGGU_, and qtRNA^His^_AGGA_ stitched together) suppression of cognate quadruplet codons at H148, G174, and S202 (**a**), H148, G174, and E213 (**b**), H148, S202, and E213 (**c**), and G174, S202, and E213 (**d**).

**
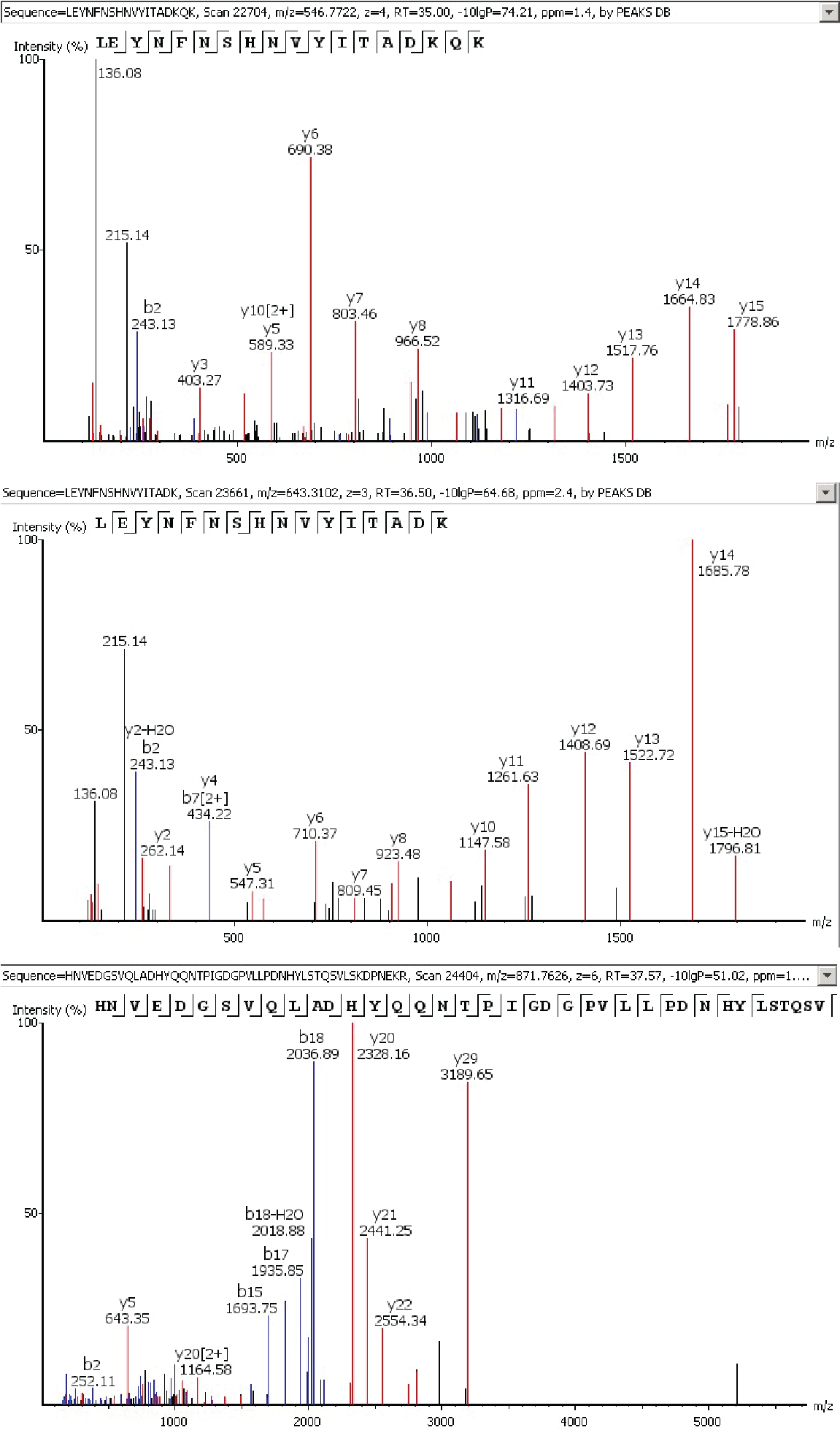
**

**Supplementary Figure 16 | LC-MS/MS analysis of qtRNA scaffold translating four quadruplet codons at positions throughout sfGFP.** Mass spectra of sfGFP fragments resulting from qtRNA scaffold #2 (composed of qtRNA^Gly^_GGGG_, qtRNA^Ser^_UAGA_-Evo3, qtRNA^Glu^_CGGU_, and qtRNA^His^_AGGA_ stitched together) suppression of cognate quadruplet codons at H148, G174, S202 and E213. Multiple peptides were observed in some cases and are shown for completeness. Summary LC-MS/MS results are reported in **Supplementary Table 8**.

**
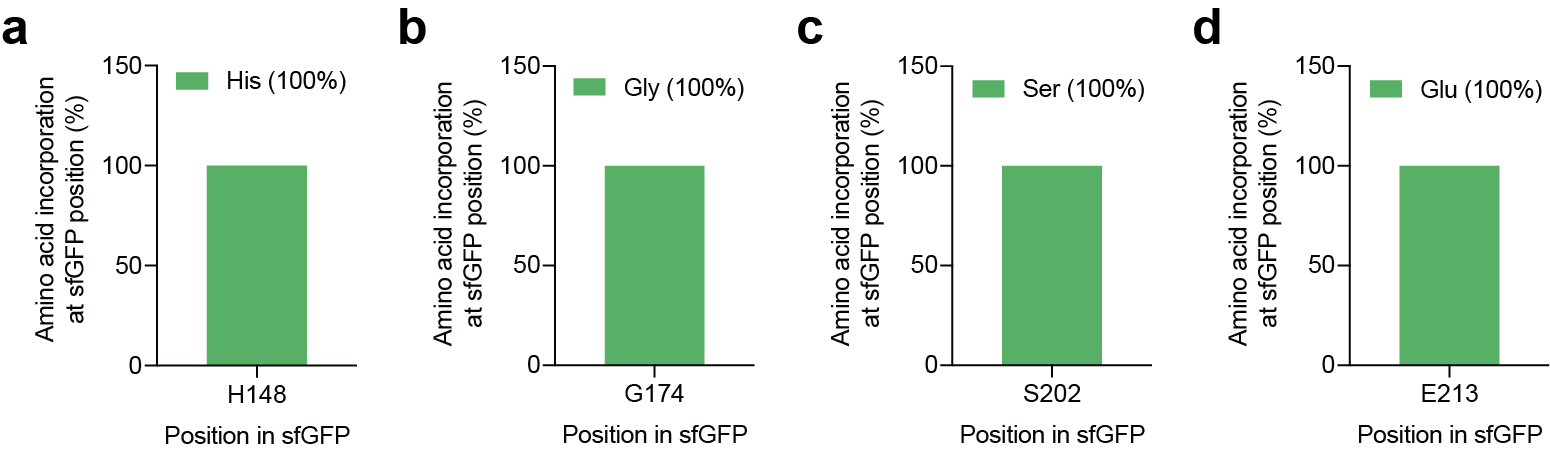
**

**Supplementary Figure 17 | Amino acid incorporation analysis corresponding to translation of four quadruplet codons in sfGFP**. Amino acid composition analysis of qtRNA scaffold #2 (composed of qtRNA^Gly^_GGGG_, qtRNA^Ser^_UAGA_-Evo3, qtRNA^Glu^_CGGU_, and qtRNA^His^_AGGA_ stitched together) suppression of cognate quadruplet codons at H148 (**a**), G174 (**b**), S202 (**c**), and E213 (**d**).

**Supplementary Table 1 | Previously reported quadruplet-decoding tRNAs discovered in bacterial isolates.** Spontaneous mutations in the tRNA which expand the anticodon by 1 base enable the decoding of quadruplet codons. Differences between the natural codon and the suppressed quadruplet codon are shown in red. AA: amino acid.


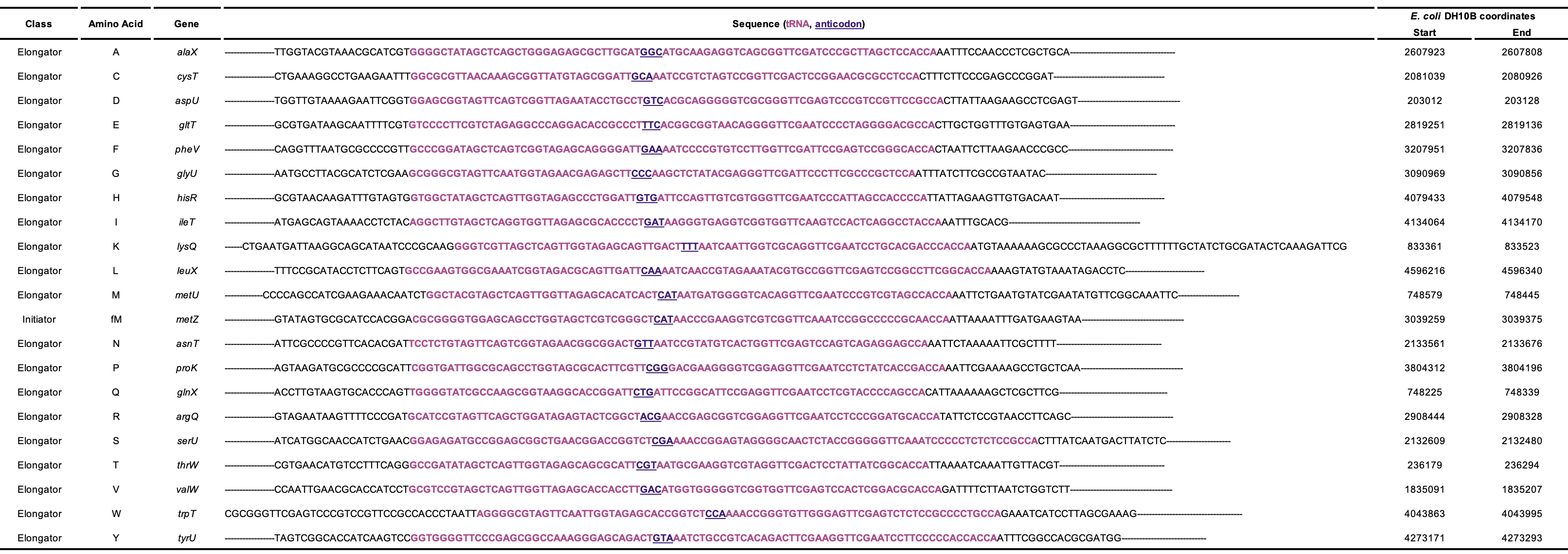


**Supplementary Table 2 | Sequences of all natural E. coli tRNA scaffolds used for qtRNA engineering.** In all cases, tRNA sequences are shown in magenta, and the anticodon is shown in purple. Flanking sequences (black) were included in vector design to ensure efficient qtRNA maturation. All coordinates derive from *E. coli* DH10B genome.

**Supplementary Table 3 | Doubling time analysis for all natural, engineered, and evolved qtRNAs.** All doubling time analyses used S3489 cells with tRNA expression plasmids encoding the shown tRNA under induced conditions. Data represents the mean and standard deviation of 4 - 8 biological replicates.

**Supplementary Table 4 | Amino acid abundance at position Y151 of sfGFP in response to UAGA quadruplet codon translation.** Mutations are indicated for each variant using universal tRNA nomenclature. AA: amino acid.

**Supplementary Table 5 | Strain doubling time analysis.** Orthogonal qtRNA expression plasmids or an engineered qtRNA scaffold were used to quantify cellular burden under uninduced and induced conditions. Data represents the mean and standard deviation of 4 - 12 biological replicates.

**Supplementary Table 6 | Sequences of multicisronic tRNA scaffolds.** Endogenous tRNA sequences are highlighted in magenta and flanking sequences are shown in black. All coordinates derive from *E. coli* MG1655 genome.

**Supplementary Table 7 | Sequences of multicisronic qtRNA scaffolds.** All qtRNAs are visualized in magenta, with their anticodons underlined in purple. Flanking sequences (black) were included in vector design to ensure efficient qtRNA maturation. qtRNA order in each scaffold is as follows: qtRNA^Gly^_GGGG_, qtRNA^Ser^_UAGA_-Evo3, qtRNA^Glu^_CGGU_, then qtRNA^His^_AGGA_

**Supplementary Table 8 | Summary LC-MS/MS results.**

**Supplementary References**

1. Lajoie, M.J. *et al.* Genomically recoded organisms expand biological functions. *Science* **342**, 357-360 (2013).

2. Johnson, D.B.F. *et al.* RF1 knockout allows ribosomal incorporation of unnatural amino acids at multiple sites. *Nature Chemical Biology* **7**, 779-786 (2011).

3. Neumann, H., Wang, K., Davis, L., Garcia-Alai, M. & Chin, J.W. Encoding multiple unnatural amino acids via evolution of a quadruplet-decoding ribosome. *Nature* **464**, 441-444 (2010).
