# Supplementary material for: Multiplex suppression of four quadruplet codons via tRNA directed evolution
Source: Nat Commun. 2021 Sep 29;12:5706. doi: 10.1038/s41467-021-25948-y (PMC8481270; doi:10.1038/s41467-021-25948-y)
Supplement: Supplementary file 2 — Reporting Summary [file 41467_2021_25948_MOESM2_ESM.pdf]

nature portfolio

Corresponding author(s): Ahmed Badran

Last updated by author(s): Sep 8, 2021

Reporting Summary

Nature Portfolio wishes to improve the reproducibility of the work that we publish. This form provides structure for consistency and transparency in reporting. For further information on Nature Portfolio policies, see our Editorial Policies and the Editorial Policy Checklist.

Statistics

For all statistical analyses, confirm that the following items are present in the figure legend, table legend, main text, or Methods section.

n/a	Confirmed	
☐	☒	The exact sample size (n) for each experimental group/condition, given as a discrete number and unit of measurement
☒	☐	A statement on whether measurements were taken from distinct samples or whether the same sample was measured repeatedly
☒	☐	The statistical test(s) used AND whether they are one- or two-sided<br>*Only common tests should be described solely by name; describe more complex techniques in the Methods section.*
☒	☐	A description of all covariates tested
☒	☐	A description of any assumptions or corrections, such as tests of normality and adjustment for multiple comparisons
☐	☒	A full description of the statistical parameters including central tendency (e.g. means) or other basic estimates (e.g. regression coefficient) AND variation (e.g. standard deviation) or associated estimates of uncertainty (e.g. confidence intervals)
☒	☐	For null hypothesis testing, the test statistic (e.g. F, t, r) with confidence intervals, effect sizes, degrees of freedom and P value noted<br>*Give P values as exact values whenever suitable.*
☒	☐	For Bayesian analysis, information on the choice of priors and Markov chain Monte Carlo settings
☒	☐	For hierarchical and complex designs, identification of the appropriate level for tests and full reporting of outcomes
☒	☐	Estimates of effect sizes (e.g. Cohen's d, Pearson's r), indicating how they were calculated

Our web collection on statistics for biologists contains articles on many of the points above.

Software and code

Policy information about availability of computer code

Data collection	R2R (version 1.0.6) was used to generate tRNA diagrams; R2R is free software available from https://sourceforge.net/projects/weinberg-r2r/. ClarioSTAR plate reader (running Reader Control 5.21 R2, BMG Labtech) and Spark plate reader (running SparkControl v2.3, Tecan) were used to generate luminescence and fluorescence data.
Data analysis	GraphPad Prism (version 8) was used for plotting and data analysis, including calculation of means and standard deviations. The Growthcurver package (version 0.3.1) in RStudio (version 3.5.2) was used to calculate doubling times from growth curves. PEAKS Studio X (Bioinformatics Solutions) was used to analyze LC-MS/MS fragmentation spectra.

For manuscripts utilizing custom algorithms or software that are central to the research but not yet described in published literature, software must be made available to editors and reviewers. We strongly encourage code deposition in a community repository (e.g. GitHub). See the Nature Portfolio guidelines for submitting code & software for further information.

Data

Policy information about availability of data

All manuscripts must include a data availability statement. This statement should provide the following information, where applicable:

- Accession codes, unique identifiers, or web links for publicly available datasets
- A description of any restrictions on data availability
- For clinical datasets or third party data, please ensure that the statement adheres to our policy

The data found in figures: 1b-e, 2a, 2d-g, 3a-c, 4a-b, 4d, 5a, 5d-f, 5h-k, and supplementary figures: 1, 2a-b, 5, 8, 9, 12, and 13 are available in the associated Source Data File. Select plasmids have been deposited to Addgene (deposit ID 77424, material ID #134787-134808 and 134812-134814) and uploaded to Benchling (see Extended Supplement for links). Remaining plasmid maps will be provided upon request.

Field-specific reporting

Please select the one below that is the best fit for your research. If you are not sure, read the appropriate sections before making your selection.

☒ Life sciences ☐ Behavioural & social sciences ☐ Ecological, evolutionary & environmental sciences

For a reference copy of the document with all sections, see nature.com/documents/nr-reporting-summary-flat.pdf

Life sciences study design

All studies must disclose on these points even when the disclosure is negative.

Sample size	Minimal sample size (generally n = 8) was determined by the dimensions of a 96-well plate. Convention in similar reports is n = 3, so we deem this number to be appropriate. Sample size is always reported in figure legends when appropriate.
Data exclusions	No data were excluded from the analyses.
Replication	For all experiments, the number of replicates is indicated in the figure or table legend.
Randomization	This study utilized clonal E. coli for all experiments, therefore randomization was not performed.
Blinding	Blinding was not performed in this study as all data are described within it, eliminating any potential for biased exclusion of data.

Reporting for specific materials, systems and methods

We require information from authors about some types of materials, experimental systems and methods used in many studies. Here, indicate whether each material, system or method listed is relevant to your study. If you are not sure if a list item applies to your research, read the appropriate section before selecting a response.

Materials & experimental systems

n/a	Involved in the study
☒	☐ Antibodies
☒	☐ Eukaryotic cell lines
☒	☐ Palaeontology and archaeology
☒	☐ Animals and other organisms
☒	☐ Human research participants
☒	☐ Clinical data
☒	☐ Dual use research of concern

Methods

n/a	Involved in the study
☒	☐ ChIP-seq
☒	☐ Flow cytometry
☒	☐ MRI-based neuroimaging
